# The Transcriptome and Metabolome Reveal Stress Responses in Sulfur-Fumigated Cucumber (*Cucumis sativus* L.)

**DOI:** 10.3389/fpls.2021.778956

**Published:** 2021-11-12

**Authors:** Juan Liu, Yang Gao, Feifei Gong, Feifan Hou, Zhipeng Zhang, Xiaojing Cheng, Wei Du, Lingling Zhang, Jinyao Wang, Jin Xu, Guoming Xing, Xiuping Kang, Sen Li

**Affiliations:** ^1^College of Horticulture, Shanxi Agricultural University, Jinzhong, China; ^2^Collaborative Innovation Center for Improving Quality and Increase of Protected Vegetables in Shanxi Province, Jinzhong, China

**Keywords:** cucumber, sulfur fumigation, stress response, transcriptome, metabolome

## Abstract

Sulfur (S) fumigation is a commonly used sterilization method in horticultural facilities against fungal diseases. S fumigation damaged cucumber leaves, although the response mechanism is unclear. This study analyzes the growth, transcriptome, and metabolomic profiles of young and mature leaves, ovaries, and commercial cucumber fruits to decipher the mechanism of cucumber stress response under S fumigation. S fumigation significantly changed the photosynthetic efficiency and reactive oxygen species (ROS) in leaves, but not fruit development, fruit mass, and peel color. Transcriptome analysis indicated that S fumigation strongly regulated stress defense genes. The weighted gene co-expression network analysis revealed that S fumigation regulated *ASPG1*, *AMC1* defense genes, *LECRK3*, and *PERK1* protein kinase. The abscisic acid (ABA)-mediated model of regulation under S fumigation was constructed. Metabolome analysis showed that S fumigation significantly upregulated or downregulated the contents of amino acids, organic acids, sugars, glycosides, and lipids (VIP > 1 and *P*-value < 0.05). The opposite Pearson’s correlations of these differential metabolites implied that cucumber had different metabolic patterns in short-term and long-term S fumigation. Besides, the elevated levels of proline and triglyceride indicated that stress-responsive mechanisms existed in S-fumigated cucumber. Moreover, the comprehensive analysis indicated that S fumigation elevated secondary S-containing metabolites but decreased sulfate absorption and transportation in cucumber. Overall, our results provided a comprehensive assessment of S fumigation on cucumber, which laid the theoretical foundation for S fumigation in protected cultivation.

## Highlights

- The effects of sulfur (S) fumigation on the growth of cucumber in protected cultivation were systematically and comprehensively analyzed, which were significantly greater in leaves than in fruits.
- Sulfur fumigation caused the accumulation of ROS and the activation of the abscisic acid (ABA) signal transduction pathway.
- Sulfur fumigation increased the content of secondary S metabolites and decreased the absorption and assimilation of sulfate.

## Introduction

Cucumber (*Cucumis sativus* L.) is a widely cultivated and popular vegetable worldwide (Li et al., 2019). Cucumbers are generally cultivated in protective environments with low light, high humidity, and low temperatures (Singh et al., 2017), conducive for breeding and spreading various fungal diseases. Sulfur (S) is an inexpensive and effective fungicide for cucumber (Keinath and DuBose, 2012). For over 100 years, S has been used to control molds and insects (Chuanzhi et al., 2020). S fumigation with temperature-controlled fumigators is a commonly used sterilization method in horticultural facilities (West and Menzies, 2002). Previous studies demonstrated that S fumigation effectively prevents breeding and spreading powdery mildew, angular spot, and downy mildew in horticultural production (Koller, 2011; Gardner-Gee, 2013; Wright et al., 2015). However, the S sterilization mechanism is unknown. The current hypothesis suggests that fine particles formed by heating S at high temperatures (190°C) perform Brownian motion in the air, penetrate the fungus, and inhibit mitochondrial respiration (Cooper and Williams, 2004; Raymond and Brown, 2015). There is multisite contact activity in S action to make the lower risk of pathogen resistance (Raymond and Gillian, 2014; Branham et al., 2020).

Sulfur is an essential mineral required for plant growth and development (Astolfi et al., 2004; Jiang et al., 2017). S is a structural constituent of vitamins, coenzymes, and prosthetic groups (Jost et al., 2005; Ausma and De Kok, 2020), including ferredoxin (Gigolashvili and Kopriva, 2014) and phytochelatins (Ha et al., 1999; Reich et al., 2016). Many S-containing compounds, including the thioproteins, glucosinolates, defensins, and glutathione, are directly or indirectly linked with plant defense against pathogenic microorganisms (Hell, 1997). Depending on the S-containing compounds, S fumigation enhances innate host defenses (Cooper and Williams, 2004). For instance, S-nutrition increased the resistance of *Brassica napus* and grapes against *Pyrenopeziza brassicae* (Burandt et al., 2001) and mildew (Schnug et al., 1995), respectively. Whether the fumigated S can be converted into S-containing compounds in the plant has a reference for evaluating the application value of S fumigation.

Although widely used in various horticultural plants, S fumigation toxicity causes leaf yellowing and necrosis (Branham et al., 2020). In the greenhouse, vaporized S caused melon to develop high S sensitivity symptoms 24 h after application (Keinath and DuBose, 2012). Additionally, S fumigation chemically transforms various biologically active ingredients, producing characteristic sulfates and sulfite derivatives (Wang et al., 2014; Kang et al., 2018). S fumigation changed the chemical characteristics of ginger by triggering chemical transformations of certain original components (Cheng et al., 2018). Therefore, the edible safety of S fumigation for cucumber cultivation requires comprehensive assessment.

This study investigates the effects of S fumigation on cucumber growth by integrating physiological, transcriptomic, and metabolomic analyses. The core molecular mechanism and key metabolic pathways were studied to understand the stress response mechanism under S fumigation. The results elucidate the regulatory network in S-fumigated cucumber and a theoretical basis for the safety evaluation of S fumigation.

## Materials and Methods

### Plant Growth and Sulfur Exposure

The *9930* cultivated cucumber inbred lines, typical of North China fresh market type cucumber, were used for this study. Cucumbers were grown in growth chambers under 16 h light/8 h dark at 28°C day/18°C night to S fumigation and control treatment, respectively. There were 20 cucumber seedlings in a growth chamber. The cucumber plants were S fumigated using 2 h/day, 0.02 g/day/m^3^ at 40-days-old, where cucumber seedlings entered the rapid growth phase (Gómez et al., 2003).

Physiological, transcriptomic, and metabolomic data were collected from three biological duplicates in cucumber fruit and leaf with developmental stages. Each biologically duplicated sample pool was collected from five cucumber plants. The treatments included ovary (0 days post-pollination, F0), commercial fruit (12 days post-pollination, F12), young leaf (ovary derived leaf, L0), and mature leaf (commercial fruit-derived leaf, L12). Supplementary Table 1 shows the specific information of sampling sites.

### Chlorophyll Contents and Chlorophyll Fluorescence Parameters

The chlorophyll (Chl) contents and Chl fluorescence parameters were measured in non-fumigated and S-fumigated L0 and L12, respectively. In brief, the Chl were extracted from 1.0 g of the fresh leaves using 95% ethanol for 24 h. The Chl extracts of at least three replicates were measured at 470, 649, and 665 nm wavelengths using the SPECORD 210 PLUS spectrophotometer (Analytik Jena GmbH, Jena, Germany).

Leaves were dark-adapted for 30 min using a pulse amplitude modulated Mini-PAM fluorometer (Heinz Walz, Effeltrich, Germany), and Chl fluorescence parameters were measured in eight replicates as previously described (Li et al., 2021). The minimum (F0) and maximum fluorescence (Fm) of dark-adapted leaves, actual (F), and maximum fluorescence of light-adapted leaves (Fm′) were evaluated. The variable to maximum fluorescence ratio (Fv/Fm) was calculated as (Fm − F0)/Fm, whereas the effective quantum yield (ΦPSII) was calculated as (Fm′ − F)/Fm′.

### Measurement of Enzyme Activities of Superoxide Dismutase, Peroxidase, and Malondialdehyde Contents

The activities of antioxidant enzymes, i.e., superoxide dismutase (SOD) and peroxidase (POD), and contents of the organic compound malondialdehyde (MDA) were determined in L0 and L12 under non-fumigated and S-fumigated treatments, respectively. The activities of SOD and POD were determined using a superoxide dismutase assay kit (Solarbio, Beijing, China) and a peroxidase assay kit (Solarbio, Beijing, China), respectively. The malondialdehyde assay kit (Solarbio, Beijing, China) measured the MDA contents. In brief, collected leaves were ground using liquid nitrogen, and SOD, POD, and MDA were extracted using the extraction buffer. The extracts were centrifuged at 12,000 × *g* for 10 min, and the supernatants were then measured for SOD, POD activities, and MDA contents using the multimode microplate reader (Tecan, Switzerland) (Yin et al., 2018). Each indicator had three replicate measurements.

### Determination of Fruit Development and Quality

Fruits were collected from 0, 3, 6, 9, and 12 days post-pollination to measure fruit development using the ratio of fruit length (*L*) and diameter (*D*). The fruit quality was evaluated by the fruit mass, peel color, and total soluble solids (TSS, %). At F12, fruit mass was measured with 30 duplicates using an analytical balance (0.01 g accuracy). The peel color of eight fruits was determined using a CR-10 Plus model colorimeter (Minolta Camera, Osaka, Japan) in the CIELAB color space. Meanwhile, the TSS (%) was determined by three fruits using a digital handheld PAL-1 pocket refractometer (Atago, Tokyo, Japan) (Yun et al., 2013).

### RNA Sequencing

The total RNA was extracted from three biological replicates of L0, L12, F0, and F12 using the mirVana miRNA Isolation Kit (Ambion, Austin, TX, United States). The RNA integrity was determined using the Agilent 2100 Bioanalyzer (Agilent Technologies, Santa Clara, CA, United States). Then, cDNA libraries were prepared from 4 μg of total RNA using the TruSeq Stranded mRNA LT Sample Prep Kit (Illumina, San Diego, CA, United States). The prepared cDNA libraries were sequenced on the Illumina HiSeq X Ten platform, and 150 bp paired-end reads were generated by the OE Biotech Co., Ltd. (Shanghai, China). Sequenced reads were quality assessed, and low-quality sequences were removed. Cleaned reads were mapped to the Chinese long cucumber genome version 3^1^ using HISAT2 (Kim et al., 2015). The read counts were estimated as the number of fragments per kilobase of the transcript sequences per million base pairs mapped (FPKM) values of expressed genes. The differentially expressed genes (DEGs) were identified using DESeq (2012) in R package (Anders and Huber, 2013) with false discovery rate (FDR) < 0.05 and | log2(Fold Change)| > = 1 (Ni et al., 2021).

### Enrichment Analysis of Differentially Expressed Genes and Weighted Gene Co-expression Network Analysis for Identifying Correlated Gene Networks

Gene ontology (GO) enrichment analysis of DEGs was performed using R (version 4.0.4) based on the hypergeometric distribution. The weighted gene co-expression network analysis (WGCNA) was performed using R with default parameters to identify co-expressed gene modules. The adjacency matrix was constructed using Pearson’s coefficients between genes and transformed into the topological overlap matrix. Clustered genes were grouped into modules according to the expression patterns. The WGCNA package associated the phenotype data with the classified modules. The gene significance (GS) and module membership (MM) of each node and the weight of node-to-node were calculated using the WGCNA package in each module. The genes of the top 10% GS and MM combined with differential expression were selected. The information on these genes was extracted from the cucumber (Chinese Long) version 3 genome. Then, hub genes within each network were obtained according to the maximal clique centrality (MCC) method, and the module network data were visualized using Cytoscape version 3.7.1 (Shanno et al., 2003).

### Validation of Hub Genes Using Quantitative Real-Time PCR

The gene expression of three independent biological replicates per sample was determined using quantitative real-time PCR (RT-qPCR). The total RNA was extracted using the RNAprep Pure Plant Kit (TIANGEN, Beijing, China), and cDNA was synthesized using a PrimeScript^TM^ RT Reagent Kit (Takara, Tokyo, Japan) with reverse transcriptase, following the instructions of the manufacturer. The hub genes were selected for RT-qPCR validation as previously described (Qin et al., 2021). The reactions were normalized using the cucumber actin gene (CuGenDB name: CsaV3_6G041900). Specific primer pairs were designed for specific unigenes and actin (Supplementary Table 2). The RNA level was expressed relative to the actin gene expression level following the 2^–ΔΔCT^ method.

### Metabolite Analysis by Ultra-Performance Liquid Chromatography and Tandem Mass Spectrometry

The same RNA-seq samples from L0, L12, F0, and F12 were used for metabolite analysis. The OE Biotech Co., Ltd. (Shanghai, China) prepared samples, extracted, identified, and quantified metabolites. In brief, ∼80 mg of tissue was transferred into an Eppendorf tube containing 20 μl internal standard (2-chloro-L-phenylalanine in methanol, 0.3 mg/ml) and 1 ml extraction solvent with methanol/H_2_O (4/1, v/v). The mixture was ground using an automatic high-throughput tissue crusher at 60 Hz, −20°C for 2 min. The extract was sonicated at 50 Hz, −20°C for 30 min, centrifuged at 13,000 rpm, and 4°C for 10 min.

The supernatant was analyzed using the ultra-performance liquid chromatography (UPLC) Triple TOF system (AB SCIEX, Framingham, MA, United States). The positive and negative modes were the ACQUITY UPLC BEH C18 column (1.7 μm, 2.1 × 100 mm) (Waters Corporation, Milford, MA, United States). Water (containing 0.1% formic acid) was the mobile phase A, and acetonitrile (containing 0.1% formic acid, v/v) was the mobile phase B. The flow rate was 0.4 ml/min, and the column temperature was 45°C. The quality control (QC) samples were used for internal standard (every 10 samples) throughout the analytical run. The ultra-performance liquid chromatography and tandem mass spectrometry (UPLC-MS/MS) raw data were analyzed using the Progenesis QI software (Waters Corporation, Milford, MA, United States), based on public^2^ ^3^ and self-built databases.

### Identification of Significantly Changed Metabolites

The metabolic alterations among experimental groups were visualized using the principal component analysis (PCA) and (orthogonal) partial least-squares-discriminant analysis [(O)PLS-DA] *via* online resources^4^. The ellipse was defined as 95% CI of the modeled variation in the score plot. The variable importance in the projection (VIP) ranked the overall contribution of each variable to the OPLS-DA model. Variables with VIP > 1.0 were considered relevant for group discrimination. The differential metabolites were selected based on the combination of statistically significant threshold VIP values from the OPLS-DA model and the *P*-values from the two-tailed Student’s *t*-test of normalized peak areas. Metabolites with VIP > 1.0 and *P* < 0.05 were considered significantly changed. Meanwhile, the bidirectional orthogonal projections to latent structures (O2PLS) were used to integrate metabolome and transcriptome data *via* online resources^5^.

## Results

### Sulfur Fumigation Altered Chlorophyll Fluorescence and the Antioxidative Capacity of Cucumber Leaves

**FIGURE 1:**
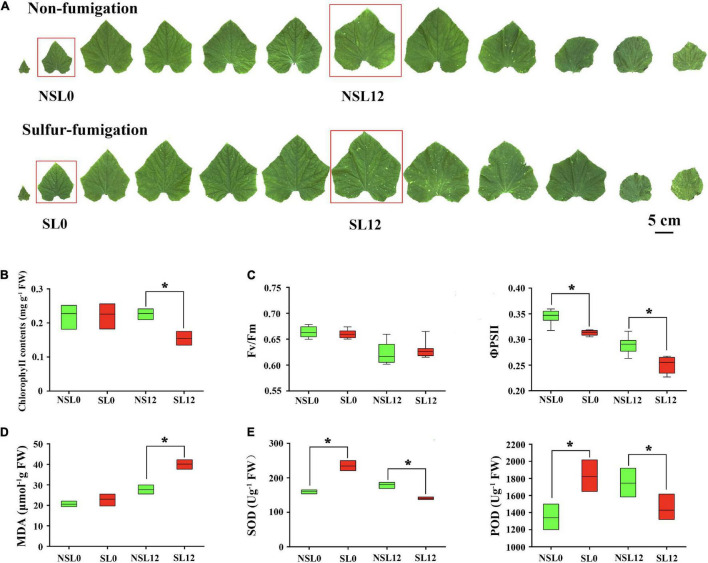
Effects of sulfur (S) fumigation on **(A)** the appearance of leaves, **(B)** chlorophyll contents, **(C)** Fv/Fm and ΦPSII, **(D)** MDA, and **(E)** SOD and POD. * indicates significant difference at *P* < 0.05, according to Duncan’s test.

There were no obvious phenotypic changes in cucumber seedlings at 40 days after sulfur fumigation (Figure 1A). S fumigation decreased Chl contents (*P* < 0.05) in L12 (Figure 1B) and significantly reduced the ΦPSII of leaves (*P* < 0.05). However, S fumigation slightly but insignificantly decreased the Fv/Fm value (Figure 1C). These results indicated that the effects of S fumigation on the photosynthetic system are greater in L12, compared with L0. Meanwhile, S fumigation increased SOD and POD activities in L0, whereas the activities decreased in L12 (*P* < 0.05) (Figure 1E). Therefore, the antioxidant enzyme activities in cucumber leaves first increased then decreased with S fumigation. Moreover, the MDA contents in L12 significantly increased (*P* < 0.05), but L0 only showed slight differences (Figure 1D).

### Effects of Sulfur Fumigation on Cucumber Fruit Development and Quality

**FIGURE 2:**
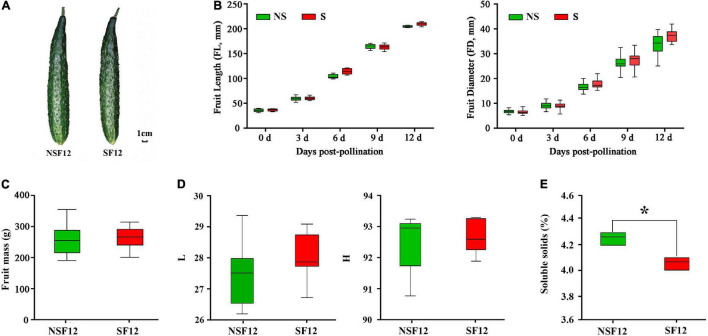
The changes in fruit quality in non-fumigated and S-fumigated treatments. **(A)** The appearance at F12. **(B)** The changes in length and diameter during fruit development. **(C)** The fruit mass at F12. **(D)** The peel luminosity and hue angle at F12. **(E)** The content of soluble solids in F12. * indicates significant difference at *P* < 0.05, according to Duncan’s test.

Sulfur fumigation had no significant impact on fruit phenotype (Figure 2A) and on fruit development (Figure 2B). The single fruit mass and the peel color of F12 were not significantly different (Figures 2C,D). Moreover, S fumigation significantly reduced the TSS content in F12 (*P* < 0.05) (Figure 2E). Overall, S fumigation did not affect cucumber development and appearance but reduced the nutritional value of commercial fruits.

### The Transcriptome Expression in Cucumber Leaves and Fruits After Sulfur Fumigation

**FIGURE 3:**
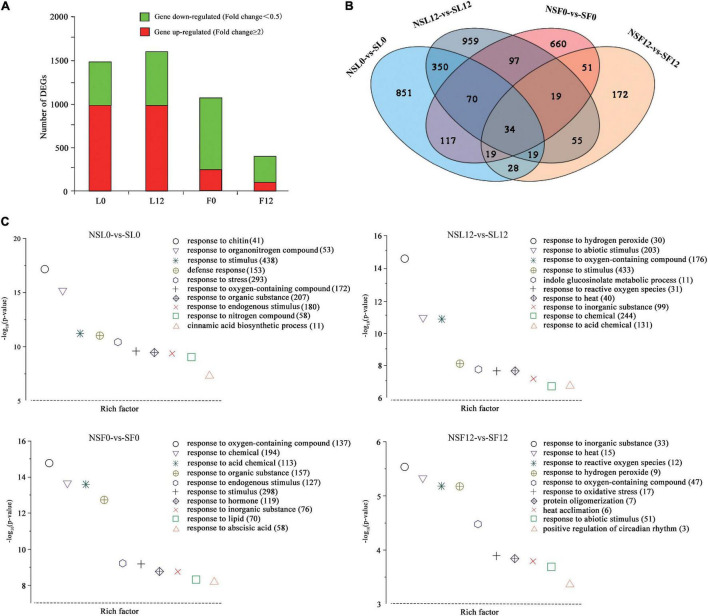
Overview of cucumber transcriptome responses to S fumigation. **(A)** The number of individual transcripts significantly upregulated or downregulated at L0, L12, F0, and F12. **(B)** Venn diagram illustrating the number of differentially expressed genes (DEGs) upregulated or downregulated by S fumigation at L0, L12, F0, and F12. **(C)** Gene ontology (GO) enrichment analysis of DEGs in the cucumber transcriptome at L0, L12, F0, and F12. Data were visualized using a scatter diagram with *P*-value levels indicated by −log10 (*P*-value) and an enrichment factor indicative of individual terms. Values in parentheses represent the number of DEGs in each term.

A total of 157.27 Gb cleaned transcriptome reads with 94.74–95.28% of bases scoring Q30 was generated from 24 samples, including three biological replicates per sample (L0, L12, F0, and F12) (Supplementary Table 3). Transcriptome analysis indicated that the leaves had more DEGs than fruits. Most of the DEGs in leaves were upregulated, while most of the DEGs in fruits were downregulated (Figure 3A). The GO enrichment analysis of the DEGs identified in L0, L12, F0, and F12 provided additional gene functions (Figure 3C). The GO terms related to abiotic stress responses in plants, including response to stimulus, response to oxygen-containing compounds, response to hydrogen peroxide, and response to reactive oxygen species (ROS), were enriched. In addition, the secondary metabolite biosynthetic processes, including organic acids and lipids, and hormone signal transduction, especially abscisic acid (ABA), were enriched in the leaves and fruits. These results indicate that S fumigation influenced more transcript expression in leaves than fruits, and the effects were significantly related to abiotic stress responses.

Notably, 34 DEGs were common between leaves and fruits (Figure 3B). Interestingly, expression patterns of 33 (of the 34-common leaf-fruit) DEGs were consistent at F0 and F12. Meanwhile, several plant abiotic stress-related genes were differentially expressed in all the groups. Several genes, including calcium uptake protein 1 homolog, mitochondrial-like (CsaV3_2G003630), octicosapeptide/Phox/Bem1p domain-containing protein kinase (CsaV3_2G024940), serine-rich protein-like protein (CsaV3_2G003620), auxin-repressed protein (CsaV3_6G050280), and NAC domain (CsaV3_3G041280), were downregulated in L0 and upregulated in L12 (Supplementary Table 4). These genes were differentially expressed in L0 and L12, reflecting different stress responses under short-term and long-term S fumigation. More importantly, inositol oxygenase (CsaV3_1G040000) and F-box protein-like (CsaV3_1G038410) were upregulated in all four groups, indicating alleviated oxidative damage (Duan et al., 2012), and induced protein degradation mediated by ubiquitination (Craig and Tyers, 1999).

### Identification of Co-expressed Gene Networks and Key Candidates

**FIGURE 4:**
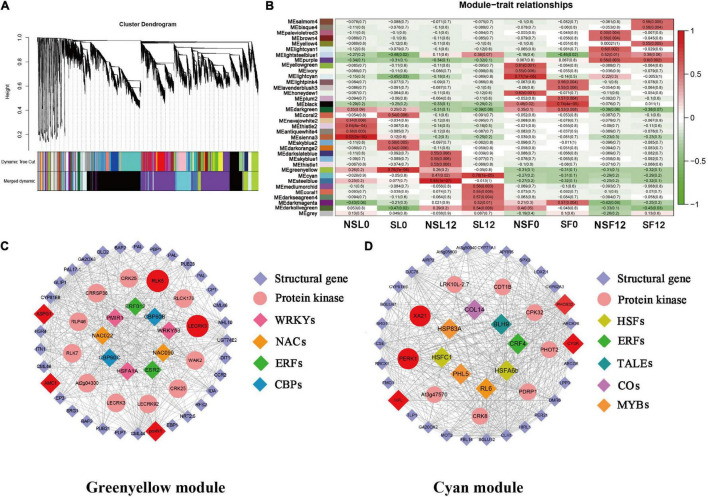
Weighted gene co-expression network analysis (WGCNA)-identified gene networks and hub genes involved in cucumber stress response after S fumigation. **(A)** Notably, 36 modules with co-expressed genes were hierarchically clustered. Each leaflet in the tree corresponds to an individual gene. **(B)** Module-trait associations based on Pearson’s correlation. The colors from green to red represent *r*^2^ values from −1 to 1. **(C)** The gene network for the green-yellow module positively correlated with SL0 (the young leaves with S fumigation) (*r*^2^ = 0.78 and *P* = 7.00E-06). **(D)** The gene network for the cyan module positively correlated with SL12 (the mature leaves with S fumigation) (*r*^2^ = 0.76 and *P* = 1.00E-05). In each network, hub genes are highlighted with enlarged spots and marked red. WRKYs, WRKY transcription factors; NAC, NAC transcription factors; ERFs, ERF transcription factors; CBPs, CBP transcription factors; HSFs, HSF transcription factors; ERFs, ERF transcription factors; TALEs, TALE transcription factors; COs, CO transcription factors; MYBs, MYB transcription factors.

Notably, 36 WGCNA modules were identified and hierarchically clustered as shown in different colors (Figure 4A). The Pearson’s correlation between modules and phenotypic traits was illustrated in the heat map (Figure 4B). Within the 36 co-expressed gene networks, the green-yellow module (1,630 genes) showed the highest positive correlation with SL0 (*r*^2^ = 0.78 and *P* = 7.00E-06). The cyan module (1,835 genes) showed genes significantly associated with SL12 (*r*^2^ = 0.76 and *P* = 1.00E-05) (Figure 4B).

The networks of selected key WGCNA-identified genes related to stress response were constructed using Cytoscape (Figures 4C,D). The co-expression network genes with respective annotations are listed in Supplementary Tables 5, 6. Multiple transcription factors, including WRKYs, NACs, ERFs, HSFs, MYBs, and protein kinases (i.e., *LECRK3*, *WAK2*, *CRK25*, *CRRSP38*, *PERK1*, *CPK32*, and *XA21*) were identified in the green-yellow and cyan modules of SL0 and SL12. Interestingly, transcripts related to phenylpropanoid biosynthesis and calcium signal transduction, including *PAL*, *CCR*, and *CP*, *CML*, were identified in the green-yellow module. *HPL* (allene oxide synthase) and *LOX* (lipoxygenase) related to jasmonic acid biosynthesis were selected in the cyan module. Therefore, S fumigation triggers various defense reactions in cucumber leaves, including phenylpropanoid biosynthesis and jasmonic acid biosynthesis.

The *LECRK3*, *RLK5*, *poxN1*, *ASPG1*, *AMC1*, *PERK1*, *XA21*, *HPL*, *CYSK*, and *PHOS32* were identified as hub genes, which included multiple protein kinases and various oxidative stress-responsive genes (Table 1). Gene *ASPG1* participates in ABA-mediated stomatal closure and improves stress resistance (Yao et al., 2012), while *AMC1* is regulated by calcium signal transduction (Zhu et al., 2020). Meanwhile, *poxN1* and *XA21* genes mediate various plant defense responses (Kim et al., 2011; Luo et al., 2012). The *CYSK* gene, related to the synthesis of S-containing amino acid (cysteine), was also identified as a hub gene. Therefore, S fumigation possibly impacts the cysteine metabolism of cucumber. The RT-qPCR expression for the hub genes was consistent with the transcriptome, confirming the reliability of the transcriptome data (Supplementary Figure 1). Overall, various defense regulatory responses such as ROS scavenging, ABA signal transduction, and calcium ion oscillation constituted the cucumber immune defense response after S fumigation.

**TABLE 1:** Hub genes selected from the co-expression modules.

Gene ID	Module	Gene symbol	Description
CsaV3_4G023950	Greenyellow module	*LECRK3*	Receptor-like protein kinase
CsaV3_1G039840	Greenyellow module	*RLK5*	Receptor-like protein kinase 5
CsaV3_7G006370	Greenyellow module	*poxN1*	Peroxidase
CsaV3_1G003730	Greenyellow module	*ASPG1*	Protein ASPARTIC PROTEASE IN GUARD CELL 1-like
CsaV3_4G004430	Greenyellow module	*AMC1*	Metacaspase-1-like
CsaV3_1G024290	Cyan module	*PERK1*	Receptor protein kinase
CsaV3_3G033940	Cyan module	*XA21*	Receptor-like protein kinase
CsaV3_7G003410	Cyan module	*HPL*	Allene oxide synthase
CsaV3_5G030130	Cyan module	*CYSK*	Cysteine synthase
CsaV3_4G001170	Cyan module	*PHOS32*	Universal stress protein A-like protein

### Abscisic Acid-Mediated Signal Transduction Improved Sulfur Tolerance in Cucumber

**FIGURE 5:**
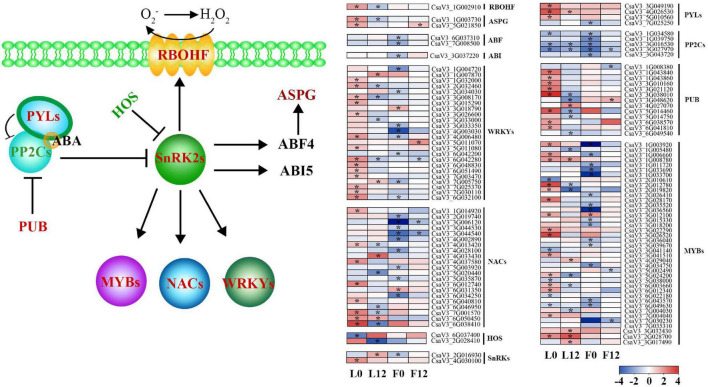
A model for the abscisic acid (ABA)-mediated stress signal transduction after S fumigation. The upregulated and downregulated genes after S fumigation are presented in blue and red boxes, respectively. The white boxes represent genes with unchanged expression. * indicates DEGs with | log2FoldChange| > 1 and *P*-value < 0.05. HOS, E3-ubiquitin-ligases; SnRK2s, SNF1-related kinases; PP2Cs, protein phosphatase 2Cs; PYLs, PYR-like genes; PUB, U-box type E3 ubiquitin ligases; RBOHF, NADPH oxidases; ASPG, aspartic protease; ABF4, ABA-responsive element binding factor 4; ABI5, ABA insensitive factor 5.

Previous analyses illustrated that S fumigation significantly affected the ABA signal transduction pathway. Inhibited by ABA receptor PYLs, the protein phosphatase 2Cs (PP2Cs) was downregulated, activating protein kinase SnRKs (Figure 5). Interestingly, the expression of these core factors was consistent in L0 and L12 under S fumigation, indicating that both short-term and long-term S fumigation significantly activated the primary ABA signal transduction pathway. Downstream of the ABA signal transduction pathway and activation of SnRKs upregulated the NADPH oxidase RBOHF profile, which promoted the accumulation of superoxide free radicals. E3-ubiquitin-ligase HOS attenuated the SnRK profile, whereas PUB inhibited the expression of PP2Cs. Furthermore, SnRKs caused differential expression of WRKYs, NACs, and MYBs. These results suggest that protein-level ABA signaling was adapted after S fumigation.

Meanwhile, other downstream defense transcripts of the ABA signal transduction pathway, except HOS, were upregulated in L0 and downregulated in L12 by S fumigation. This means that short-term and long-term S-induced stress responses are different. Moreover, the ABA signal transduction pathway was significantly downregulated in the S fumigated F0 but not F12, possibly indicating that S fumigation affected endogenous ABA metabolism in the ovary.

### Metabolic Changes in Cucumber Leaves and Fruits After Sulfur Fumigation

The metabolic profiles were identified by UPLC-MS/MS. The PCA score plots showed that the L0, L12, and F0 groups were clearly separated from the control along with the first principal component. However, there was no noticeable separation between the F12 group and the control group (Supplementary Figure 2A). To maximize the separation between groups, OPLS-DA was performed on the basis of the metabolite data. The score plots showed that L0, L12, F0, and F12 groups were clearly separated from the control (Supplementary Figure 2B). This indicated that S fumigation altered metabolite profiles in cucumber. The Pearson’s correlation coefficient between each sample was calculated to measure the repeatability and quality (Supplementary Figure 3). The one-way ANOVA results showed that L12 had more differentially expressed metabolites (DEMs) than L0, whereas F12 had fewer DEMs than F0 (Supplementary Figure 4). Overall, there were more downregulated than upregulated DEMs (Supplementary Figure 4A).

**FIGURE 6:**
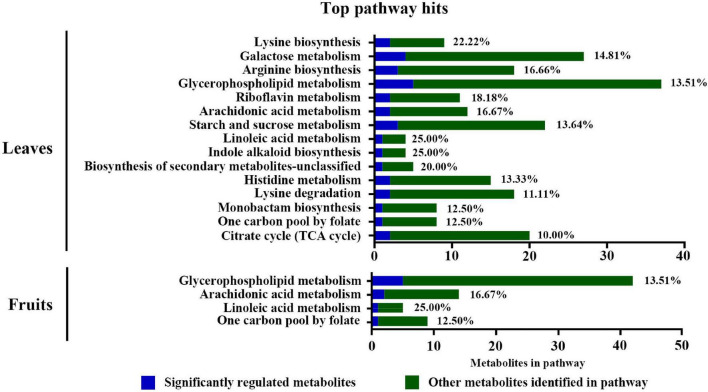
Pathway analysis of the DEMs detected in leaves and fruits under non-fumigation and S fumigation, respectively. The *x*-axis represents the number of DEMs annotated in each pathway. The blue portions represent differentially detected metabolites, while the green portions represent the other annotated metabolites in the pathway. The percentages are the ratios of blue to green. Only pathways with at least 10% significant regulation were included.

The Kyoto Encyclopedia of Genes and Genomes (KEGG) enrichment analysis showed that the glycerophospholipid metabolism, arachidonic acid metabolism, and linoleic acid metabolism were substantially enriched in leaves and fruits (Figure 6). Additionally, sucrose and starch, galactose, lysine, arginine metabolic pathways, and the TCA cycle were enriched in leaves. Supplementary Tables 6, 7 summarize the significantly changed metabolites mostly related to S fumigation in leaves and fruits. Various sugars, glycosides, organic acids, and amino acids were significantly changed in leaves and fruits (*P* < 0.05) (Supplementary Tables 7, 8). These results indicate that S fumigation regulates carbon and nitrogen metabolism in cucumbers.

The Pearson’s correlation analysis of the top 50 VIP DEMs in each group identified 21, 9, and 15 lipid metabolites in L0, L12, and F0, respectively. The lipid metabolites positively correlated with sugars (i.e., sucrose, trehalose, galactose, and mannose) (Supplementary Figures 5–7). Interestingly, the lipid metabolites from L0 and F0 positively correlated with several glycosides and negatively correlated with citric acid, maleic acid, and 17-hydroxylinolenic acid (Supplementary Figures 5, 7). However, the lipid metabolites from L12, except PA(15:0/20:3(5Z, 8Z, 11Z)) and TG(16:0/18:1(9Z)/18:1(9Z)), positively correlated with several organic acids [17-hydroxylinolenic acid, pyrrolidone carboxylic acid, and 3,4,5-trihydroxy-6-(2-hydroxy-6-methoxyphenoxy)oxane-2-carboxylic acid] and negatively correlated with several glycosides (Supplementary Figure 6). Thus, short-term and long-term S fumigation had differentially regulated cucumber metabolites.

### Sulfur Fumigation Affected Carbon and Nitrogen Metabolism in Cucumber

**FIGURE 7:**
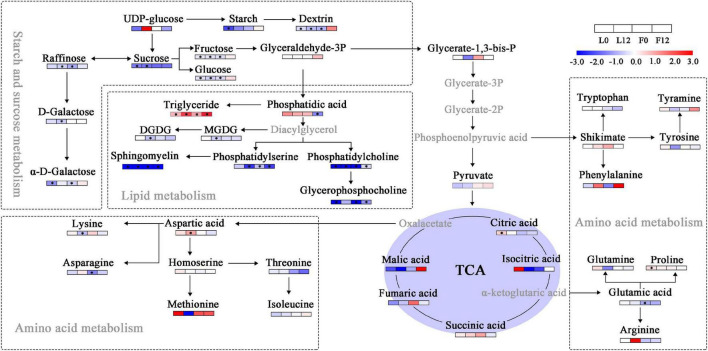
Changes in sugars, lipids, and amino acids in tricarboxylic acid cycle (TCA) metabolism. Blue and red boxes, respectively, represent metabolites with lower or higher abundance under S fumigation. The white boxes represent metabolites with unchanged abundances. The gray letters represent undetected metabolites. * indicates DEMs with *P*-value < 0.05 and VIP > 1. DGDG, digalactosyldiacylglycerol; MGDG, monogalactosyldiacylglycerol.

Citric acid, which participated in the cucumber TCA cycle, was significantly upregulated in cucumber (Figure 7). Furthermore, several sugars, including sucrose, fructose, glucose, raffinose, and galactose, significantly decreased. S fumigation significantly reduced the contents of lipids such as phosphatidic acid, phosphatidylserine, phosphatidylcholine, sphingomyelin, and glycerophosphocholine. More importantly, the contents of triglyceride and proline significantly increased under S fumigation. Triglycerides and proline are essential for peroxide loading (Feussner et al., 1997) and relieving osmotic stress (Abogadallah et al., 2003), respectively. These results proved that S fumigation significantly reduced carbon and lipid metabolism in cucumber, a factor related to the decreased leaf photosynthetic efficiency caused by S fumigation.

### Combined Transcriptome and Metabolome Analysis Revealed Changes in Sulfur Metabolism in Cucumber

**FIGURE 8:**
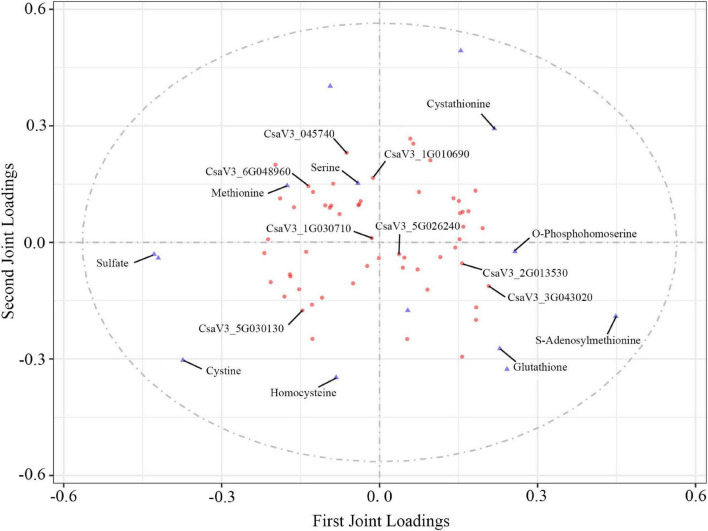
O2PLS loading plot of metabolites and transcripts involved in S-related metabolism. Transcripts (circles) and metabolites (triangles) represent individual transcript and metabolite loading values, respectively (Supplementary Tables 9, 10).

**FIGURE 9:**
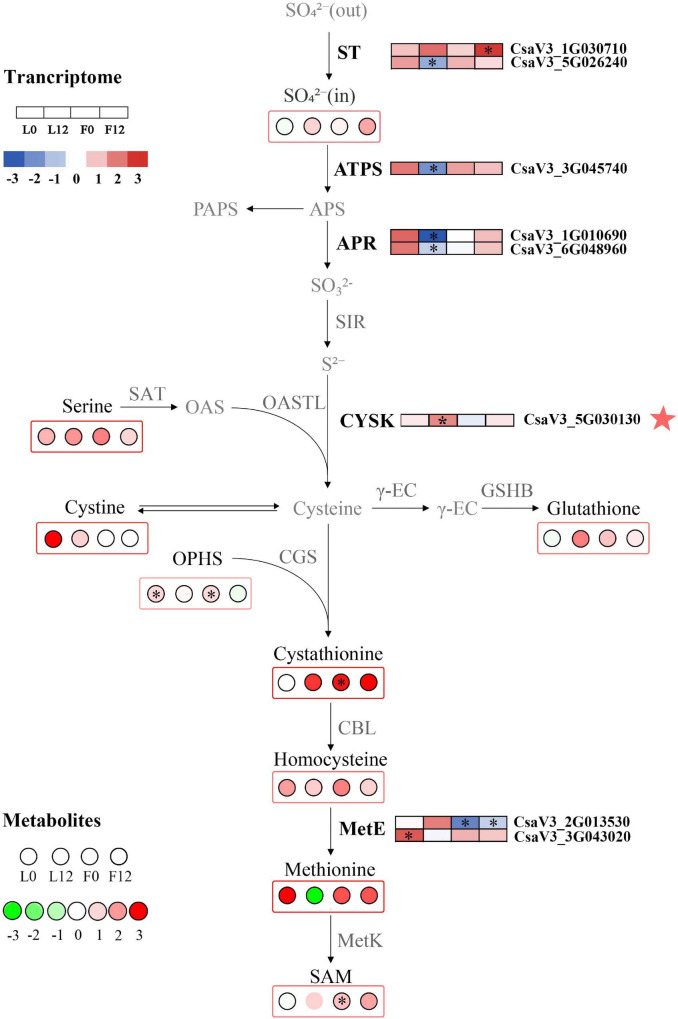
Changes in S metabolism in S fumigated cucumber. Upregulated and downregulated genes and metabolites due to S fumigation are represented by blue-to-red boxes and green-to-red circles, respectively. The white boxes or circles represent unchanged genes or metabolites. ^∗^ indicates DEGs or DEMs with | log_2_FoldChange| > 1 and *P*-value < 0.05, or VIP > 1 and *P*-value < 0.05. ST, sulfate transporters; ATPS, ATP sulfurylase; APR, adenosine phosphosulphate reductase; SIR, sulfate reductase; OASTL, O-acetylserine (thiol) lyase; SAT, serine acetyltransferase; CYSK, cysteine synthase; γ-EC, γ-glutamylcysteine synthetase; GSHB, glutathione synthetase; CGS, cystathionine γ-synthase; CBL, cysteine-S-conjugate β-lyase; MetE, L-glutamine-4-(methylsulfanyl)-2-oxobutanoate aminotransferase; MetK, S-adenosylmethionine synthetase. The red star represents the hub gene identified by WGCNA.

To explore the effects of S fumigation on the S-related metabolism of cucumber leaves and fruits, the integration of transcriptome and metabolome was performed using O2PLS. Of note, 57 transcripts and 14 metabolites were used for model construction (R^2^X = 0.694 and R^2^Y = 0.838). As shown in Figure 8, *CYSK* (CsaV3_5G030130), one of the WGCNA-identified hub genes, was positively correlated with S-containing amino acids (i.e., cystine and homocysteine). Meanwhile, strong negative correlations could be seen between glutathione and transcripts involved in sulfate transport, activation, and reduction (i.e., CsaV3_3G045740, CsaV3_1G010690, and CsaV3_6G048960). These genes were significantly downregulated in L12, which indicates that sulfate absorption and assimilation decreased under long-term S fumigation (Figure 9). Moreover, S fumigation significantly increased the contents of various S-containing metabolites, for example, cystathionine, O-phosphohomoserine (OPHS), and S-adenosylmethionine (SAM) in cucumber leaves and fruits. The contents of sulfate, serine, cystine, glutathione, and homocysteine slightly but insignificantly increased. Altogether, these results indicate that the increase of secondary S-containing metabolites in S-fumigated cucumbers may be through the conversion of non-S-containing metabolites, rather than through the enhancement of cucumber sulfate assimilation.

## Discussion

Sulfur fumigation is a common sterilization method in organic protected horticulture. However, this method may cause scorching in some plants, such as melon and tomato, where plants with excessive scorched died (Jiang et al., 2017; Branham et al., 2020). This study shows that long-term S fumigation did not significantly affect the phenotype, Chl contents, and Fv/Fm of cucumber *9930*, suggesting that *9930* is an S-tolerant cucumber variety. Meanwhile, the H_2_O_2_ content and the lipid peroxidation level were determined in this study. Generally, oxidative stress leads to changes in antioxidant enzyme activities (Zhao et al., 2021). In this study, the higher activities of SOD and POD, along with the slight increase of MDA, indicate that mild oxidative stress seems to occur under the short-term S fumigation in this study. This mechanism would allow keeping the appropriate H_2_O_2_ levels for both signaling and oxidative protection (Ernestina et al., 2020). However, long-term S fumigation broke this balance, which was manifested by the decrease of SOD and POD activities and a significant increase of MDA. This physiological response of S fumigation is similar to the defense of plants under drought, salt, and cold stresses (Auler et al., 2021; Wei et al., 2021; Zhao et al., 2021).

Sulfur fumigation caused transcriptional and metabolic defense responses in cucumbers, but the responses were more transcriptional than metabolic. The cucumber stress response after S fumigation involved multigene responses. Genes involved in stress regulation are often regulated through coordinated expression. Therefore, correlation-based models are used to identify gene networks. In this study, the hub genes identified indicated that S fumigation triggers protein kinases, ROS scavenging, ABA signal transduction, and calcium signal transduction. For example, *ASPG1* is an ABA-signaling gene that mediates stress response by causing stomatal closure (Yao et al., 2012). Similarly, *AMC1* is involved in calcium-regulated immune defense (Zhu et al., 2020), and *XA21*, a broad spectrum receptor-like kinase resistance gene, was also upregulated by S fumigation (Luo et al., 2012; Park and Ronald, 2012). The identified gene, *CYSK*, is essential in cysteine metabolism. However, *PAL* and *CCR* genes for phenylpropanoid biosynthesis were also differentially expressed. These results suggested that multiple complex defense responses significantly contribute to the observed resistance after S fumigation.

The plant hormone, ABA, triggered by S fumigation, is essential in plant defense against stress (Chen et al., 2020). ABA changes stomatal resistance to regulate leaf transpiration rate and the expression of stress-related genes through signal transduction (Shinozaki and Yamaguchi-Shinozaki, 1997; Niu et al., 2018). The ABA-regulated SnRKs promote H_2_O_2_ production in the apoplast (Szymańska et al., 2019). The H_2_O_2_ activates Ca^2+^ channels in guard cells to maintain basic ROS levels in plants (Yang et al., 2018). This study showed that S fumigation upregulated SnRKs in leaves and affected the expression of the PYLs/PP2Cs complex. Moreover, Ca^2+^ signal transduction and POD were differentially expressed under S fumigation. These results suggest that S fumigation elicited complex signal crosstalk among ABA, ROS, and Ca^2+^ in cucumber. However, short-term S fumigation might cause subtle stress responses. In contrast, long-term S fumigation intensifies the immune response in cucumber, shown by transcript changes in the calcium signal transduction and ROS scavenging systems.

In plants, abiotic stress reduces energy through metabolite conversion (Zhu, 2016). Water deficit causes sucrose and raffinose accumulation to protect the plants from drought stress (Peters et al., 2007). However, S fumigation significantly decreased sugars such as sucrose, fructose, and raffinose in cucumber, probably due to the decreased photosynthetic efficiency after S fumigation, although the molecular mechanism remains unclear. This study indicates that proline, a free amino acid, was significantly upregulated in cucumber leaves upon S fumigation. Free amino acids regulate plant stress by maintaining stable osmotic pressure in plants (Rodriguez and Redman, 2005). However, this hypothesis needs to be further explored.

The increased SOD and POD enzyme activities in cucumber leaves denote that S fumigation caused the production of free radicals. Excessive oxygen free radicals in plants react with lipids, nucleic acids, and other metabolites, causing lipid peroxidation, membrane damage, and enzyme inactivation (Gill and Tuteja, 2010). Previous studies proved triglyceride as the main product of lipid oxidation in cucumber (Feussner et al., 1997). In this study, triglycerides were significantly upregulated in cucumber leaves and fruits after S fumigation, indicating that S fumigation caused lipid peroxidation. Additionally, various lipids were significantly downregulated by S fumigation. The metabolite correlation analysis showed that lipids were positively correlated with various sugars and glycosides and negatively correlated with several organic acids under short-term S fumigation. However, long-term S fumigation reversed this correlation trend in leaves. Studies have shown that stressed plants accumulate organic acids to improve resistance (López-Bucio et al., 2000). Therefore, the increased contents of organic acids in cucumber after short-term S fumigation enhanced resistance, while resistance was weakened by long-term S fumigation.

In this study, S fumigation provided a system for S metabolism in cucumber. This study also confirms that sulfate absorption, transport, and assimilation transcripts were significantly downregulated, and an observation similar to that in *Arabidopsis thaliana* exposed to sulfur dioxide (Zhao and Yi, 2014). In another study, the sulfate transport *APR* rapidly decreased after sulfite application in tomatoes, a strategy to maintain sulfite homeostasis and avoid sulfite accumulation (Brychkova et al., 2013). However, S fumigation increased various S-containing compound profiles. These results suggest that S fumigation does not increase S-containing compound profiles by promoting sulfate assimilation in cucumber. This specific regulatory mechanism remains to be explored. Therefore, S fumigation could be used to regulate S nutrition in cucumber.

**FIGURE 10:**
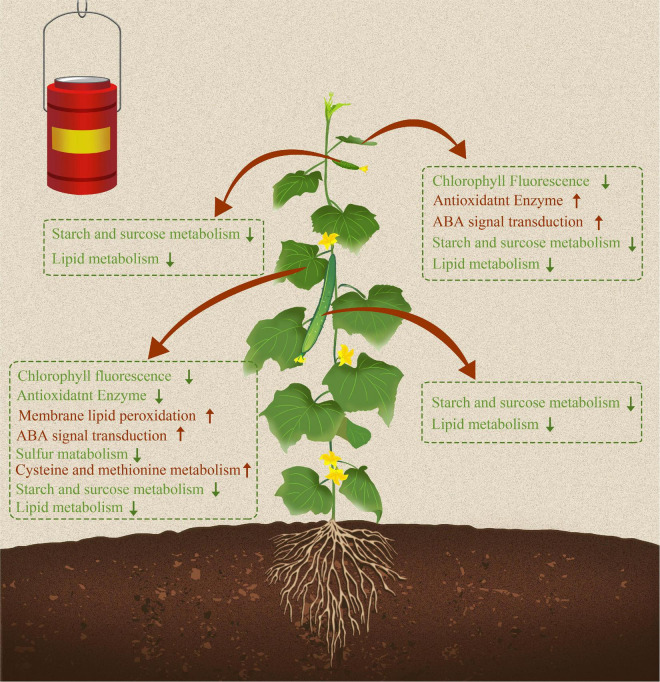
Sulfur fumigation modulated growth, transcription expression profiles, and metabolic pathways in cucumber leaves and fruits. Red and green arrows represent upregulated and downregulated physiological indicators, transcriptional regulation pathways, and metabolic pathways due to S fumigation.

Overall, both short-term and long-term S fumigation caused abiotic stress responses in leaves (Figure 10). The stress suffered by the increase with the time of S fumigation. Moreover, S fumigation damaged and changed carbon and nitrogen metabolism in cucumber. However, the damage was more significant in leaves and subtle on fruits, especially the commercial fruits. Therefore, S fumigation must be performed within the appropriate duration for effective bactericidal effects and reduced damage to plants. The timing of S fumigation is the challenge requiring further studies to facilitate the adoption of S fumigation. Additionally, future studies should focus on finding key response factors in cucumbers and illustrating how key factor enhances resistance in S-fumigated cucumber. The genetic mining of S-tolerant genes is recommended.

## Data Availability Statement

The original contributions presented in the study are publicly available. This data can be found here: National Center for Biotechnology Information (NCBI) BioProject database under accession number PRJNA765376.

## Author Contributions

JL wrote the manuscript. YG, FG, and WD analyzed the transcriptome data. FH, ZZ, and JW analyzed the metabolome data. XC and LZ carried out the physiological determination. JX and GX advised the manuscript. XK and SL supported the whole project. All authors contributed to the article and approved the submitted version.

## Conflict of Interest

The authors declare that the research was conducted in the absence of any commercial or financial relationships that could be construed as a potential conflict of interest.

## Publisher’s Note

All claims expressed in this article are solely those of the authors and do not necessarily represent those of their affiliated organizations, or those of the publisher, the editors and the reviewers. Any product that may be evaluated in this article, or claim that may be made by its manufacturer, is not guaranteed or endorsed by the publisher.

## Footnotes

1
http://cucurbitgenomics.org/organism/20

2
http://www.hmdb.ca/

3
http://www.lipidmaps.org/

4
http://www.metaboanalyst.ca/

5
https://www.omicshare.com/tools
