# Supplementary material for: The Transcriptome and Metabolome Reveal Stress Responses in Sulfur-Fumigated Cucumber (Cucumis sativus L.)
Source: Front Plant Sci. 2021 Nov 12;12:778956. doi: 10.3389/fpls.2021.778956 (PMC8636124; doi:10.3389/fpls.2021.778956)
Supplement: Supplementary file 1 [file Data_Sheet_1.docx]

**Table S1** The specific information of sampling sites

| Organ | State | Sample naming | |
| --- | --- | --- | --- |
|  |  | Sulfur fumigation | Non sulfur fumigation |
| Leaf | Young leaf | SL0 | NSL0 |
|  | Mature leaf | SL12 | NSL12 |
| Fruit | Ovary | SF0 | NSF0 |
|  | Commercial fruit | SF12 | NSF12 |

**Table S2** Primers of RT-qPCR analysis

| Gene symbol | Forward primers | Reverse primers |
| --- | --- | --- |
| *Actin* | CTCCACTCAACCCAAAGGCTA | AGAATCCAGCACGATACCAGT |
| *LECRK3* | CTTCCAGCTTCTGTTCAACCT | CATCGTGCTCAAGAATTGCC |
| *RLK5* | ATGGAAATCATTGCAGCGTCT | CCAACGAAAGCGGAATTGACC |
| *poxN1* | ACCCGCATTGGTTTCTACTCC | TCACAACCCCGAACGAAGCA |
| *ASPG1* | GCTGACGAGTAGCCTATCACC | GAGGTTCCTTCCGACAACACA |
| *AMC1* | ACATCAGAATGGCGATGCAA | CATAATCCAATGGGCAAAGCG |
| *PERK1* | AATTCGCTTGTTCCTATCCCA | TCTGTCGCCATTGCTAACTCT |
| *XA21* | TCAGATTCCGAACGAATTGTCC | TGAAAGTTGTTCCGCCCGAA |
| *HPL* | AACCAATGTTCCTCCGTCT | GGCATGAAATCACCGACCAG |
| *CYSK* | TGGAGCTGGTTTTATACCCAA | TGCTCCAGAAGAAATACCCAC |

**Table S3** RNA sequencing data and quality control information

| Sample | Raw Reads | Clean Reads | Clean Bases (G) | Q30 (%) | GC Content (%) |
| --- | --- | --- | --- | --- | --- |
| NSL0-1 | 48.18 | 47.47 | 6.62 | 95.17 | 44.88 |
| NSL0-2 | 48.54 | 47.83 | 6.69 | 95.14 | 45.05 |
| NSL0-3 | 43.52 | 42.85 | 6.00 | 95.13 | 44.93 |
| SL0-1 | 48.45 | 47.51 | 6.66 | 94.79 | 45.12 |
| SL0-2 | 47.70 | 46.90 | 6.58 | 94.93 | 45.08 |
| SL0-3 | 51.12 | 50.18 | 6.98 | 94.75 | 45.02 |
| NSL12-1 | 44.41 | 43.72 | 6.07 | 95.10 | 44.43 |
| NSL12-2 | 45.02 | 44.31 | 6.18 | 95.10 | 44.34 |
| NSL12-3 | 47.22 | 46.44 | 6.51 | 94.87 | 44.55 |
| SL12-1 | 47.87 | 47.14 | 6.66 | 92.73 | 44.78 |
| SL12-2 | 48.90 | 48.06 | 6.73 | 91.82 | 44.60 |
| SL12-3 | 51.58 | 50.80 | 7.14 | 92.33 | 44.49 |
| NSF0-1 | 44.18 | 43.94 | 6.14 | 95.09 | 44.84 |
| NSF0-2 | 45.95 | 45.25 | 6.31 | 91.57 | 44.80 |
| NSF0-3 | 42.55 | 41.95 | 5.90 | 95.21 | 44.94 |
| SF0-1 | 49.40 | 48.61 | 6.84 | 94.95 | 44.83 |
| SF0-2 | 47.89 | 47.03 | 6.55 | 95.11 | 44.95 |
| SF0-3 | 48.87 | 48.08 | 6.75 | 95.14 | 45.07 |
| NSF12-1 | 48.01 | 47.18 | 6.65 | 94.75 | 44.59 |
| NSF12-2 | 47.82 | 46.94 | 6.60 | 94.74 | 44.56 |
| NSF12-3 | 48.71 | 47.84 | 6.74 | 94.76 | 44.57 |
| SF12-1 | 47.97 | 47.25 | 6.63 | 95.23 | 44.58 |
| SF12-2 | 49.18 | 48.40 | 6.78 | 95.09 | 44.66 |
| SF12-3 | 47.40 | 46.66 | 6.56 | 95.15 | 44.60 |

**Table S4** DEGs common between L0, L12, F0, and F12 transcriptomes


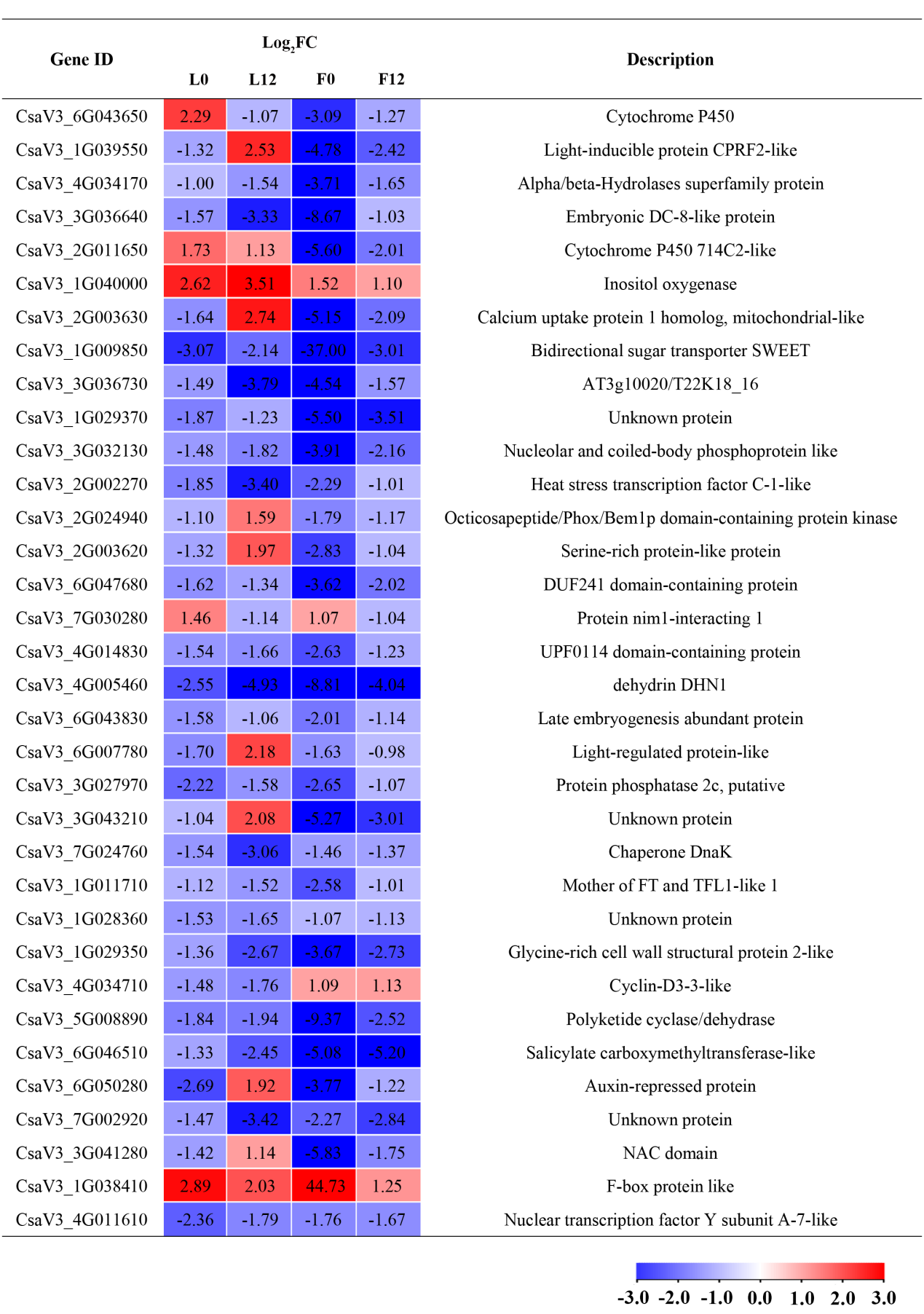


**Table S5** The key genes from the Greenyellow module identified by WGCNA

| Gene ID | Gene symbol | Description |
| --- | --- | --- |
| CsaV3_6G050100 | *HSFA1A* | Heat stress transcription factor B-2a-like |
| CsaV3_3G021980 | *WRKY53* | WRKY transcription factor, putative |
| CsaV3_6G032100 | *PMIR1* | WRKY transcription factor, putative |
| CsaV3_5G031070 | *ESR2* | Ethylene-responsive transcription factor ESR2-like |
| CsaV3_4G028360 | *ERF012* | Ethylene-responsive transcription factor ERF014 |
| CsaV3_4G006510 | *ERF061* | Ethylene-responsive transcription factor ERF061 |
| CsaV3_7G001570 | *NAC022* | NAC domain-containing protein, putative |
| CsaV3_6G050450 | *NAC073* | NAC domain-containing protein |
| CsaV3_6G038410 | *NAC090* | NAC domain-containing protein 90 |
| CsaV3_4G023950 | *LECRK3* | Receptor-like protein kinase |
| CsaV3_4G037780 | *WAK2* | Wall-associated receptor kinase 2-like |
| CsaV3_1G011130 | *CRK25* | Cysteine-rich receptor-like protein kinase |
| CsaV3_7G035130 | *At2g04300* | Protein kinase family protein |
| CsaV3_1G011160 | *CRK25* | Cysteine-rich receptor-like protein kinase 10 |
| CsaV3_4G023940 | *LECRK3* | Receptor-like protein kinase |
| CsaV3_5G026700 | *RLCK176* | Protein kinase |
| CsaV3_1G041610 | *RLP46* | LRR receptor-like serine/threonine-protein kinase GSO1 |
| CsaV3_7G003810 | *CRRSP38* | Cysteine rich receptor like kinase |
| CsaV3_7G007500 | *RLK7* | Receptor-like protein kinase HAIKU2 |
| CsaV3_1G039840 | *RLK5* | Receptor-like protein kinase 5 |
| CsaV3_7G007210 | *LECRK92* | Receptor lectin kinase |
| CsaV3_3G015240 | *CP1* | Calmodulin |
| CsaV3_1G005550 | *CML46* | Calcium-binding EF-hand family protein, putative |
| CsaV3_3G041300 | *CML46* | Calcium-binding EF hand family protein |
| CsaV3_6G034010 | *CML44* | Calcium-binding protein |
| CsaV3_7G006370 | *poxN1* | Peroxidase |
| CsaV3_6G043650 | *CYP81E8* | Cytochrome P450 |
| Gene ID | Gene symbol | Description |
| CsaV3_6G039720 | *PAL17.1* | Phenylalanine ammonia-lyase |
| CsaV3_4G002330 | *PAL* | Phenylalanine ammonia-lyase |
| CsaV3_4G002310 | *PAL* | Phenylalanine ammonia-lyase |
| CsaV3_4G002320 | *PAL* | Phenylalanine ammonia-lyase |
| CsaV3_3G014440 | *SRG1* | 2-oxoglutarate (2OG) and Fe(II)-dependent oxygenase Superfamily protein |
| CsaV3_1G003730 | *ASPG1* | Protein ASPARTIC PROTEASE IN GUARD CELL 1-like |
| CsaV3_1G036780 | *EP3* | Endochitinase EP3-like |
| CsaV3_1G011060 | *GA2OX8* | Gibberellin 2-beta-dioxygenase 8 |
| CsaV3_6G046590 | *HSR4* | ATP-dependent zinc metalloprotease FtsH-like protein |
| CsaV3_3G009190 | *BAP2* | BON1-associated protein 2-like |
| CsaV3_4G004430 | *AMC1* | Metacaspase-1-like |
| CsaV3_5G001480 | *DLO2* | Protein DMR6-LIKE OXYGENASE 2-like |
| CsaV3_7G033580 | *ITN1* | Ankyrin repeat-containing protein |
| CsaV3_3G009160 | *BAP2* | BON1-associated protein 2-like |
| CsaV3_4G024240 | *DIT1* | Dicarboxylate transporter 1, chloroplastic |
| CsaV3_1G011690 | *PBP1* | B-box zinc finger protein 32-like |
| CsaV3_3G027450 | *UGT74E2* | Glycosyltransferase |
| CsaV3_3G038010 | *PUB23* | RING-type E3 ubiquitin transferase |
| CsaV3_6G052720 | *PLP7* | Patatin |
| CsaV3_3G032850 | *CCR2* | Cinnamoyl coa reductase |
| CsaV3_3G037870 | *NHL10* | Late embryogenesis abundant protein |
| CsaV3_3G030920 | *GLIP1* | GDSL esterase/lipase 1-like |
| CsaV3_1G000950 | *RFS2* | Galactinol--sucrose galactosyltransferase |
| CsaV3_4G026380 | *EBP1* | Proliferation-associated 2G4-like protein |
| CsaV3_3G021120 | *PUB21* | Transcription factor |
| CsaV3_5G033860 | *IDA* | Protein IDA-LIKE |
| CsaV3_5G031350 | *NRT2.5* | High-affinity nitrate transporter 2.2 |

**Table S6** The key genes from the Cyan module identified by WGCNA

| Gene ID | Gene symbol | Description |
| --- | --- | --- |
| CsaV3_5G002210 | *CRF4* | Ethylene-responsive transcription factor CRF2-like |
| CsaV3_1G024170 | *HSFC1* | Heat stress transcription factor C-1-like |
| CsaV3_3G012910 | *HSFA6b* | Heat stress transcription factor A-6b |
| CsaV3_3G032430 | *PHL5* | Myb family transcription factor |
| CsaV3_2G028700 | *RL6* | Protein RADIALIS-like 4 |
| CsaV3_3G017490 | *HSP83A* | Protein REVEILLE 6-like isoform X2 |
| CsaV3_4G002870 | *COL14* | Zinc finger protein CONSTANS-LIKE 15-like |
| CsaV3_4G025530 | *BLH9* | BEL1-like homeodomain protein 9 isoform X1 |
| CsaV3_1G024290 | *PERK1* | Receptor protein kinase |
| CsaV3_4G002860 | *CPK32* | Calcium-dependent protein kinase, putative |
| CsaV3_6G049370 | *CRK8* | Cysteine rich receptor like kinase |
| CsaV3_1G006840 | *LRK10L-2.7* | Receptor-like kinase |
| CsaV3_3G033940 | *XA21* | Receptor-like protein kinase |
| CsaV3_3G033930 | *At3g47570* | Kinase-like protein |
| CsaV3_1G011070 | *PDRP1* | Pyruvate, phosphate dikinase regulatory protein |
| CsaV3_3G043220 | *CDT1B* | CDT1-like protein a, chloroplastic |
| CsaV3_7G002270 | *PHOT2* | Phototropin-2-like isoform X1 |
| CsaV3_4G023930 | *LOX2.1* | Lipoxygenase |
| CsaV3_3G035690 | *PER21* | Peroxidase |
| CsaV3_6G051310 | *SRG1* | Oxoglutarate-dependent dioxygenase 2 |
| CsaV3_6G041260 | *GA20OX2* | Gibberellin 20 oxidase 2-like |
| CsaV3_7G003410 | *HPL* | Allene oxide synthase |
| CsaV3_7G027450 | *DMR6* | Hyoscyamine 6-dioxygenase-like |
| CsaV3_6G043630 | *CYP81E8* | Cytochrome P450 |
| CsaV3_5G010170 | *At5g05600* | Leucoanthocyanidin dioxygenase-like |
| CsaV3_5G010170 | *CYP82A3* | Cytochrome P450 CYP82D47-like |
| CsaV3_1G028400 | *FMO1* | Flavin-containing monooxygenase |
| Gene ID | Gene symbol | Description |
| CsaV3_1G008980 | *CYP71A1* | Cytochrome P450 |
| CsaV3_2G033910 | *LPP3* | Lipid phosphate phosphatase 2 |
| CsaV3_5G030130 | *CYSK* | Cysteine synthase |
| CsaV3_1G010960 | *BGLU47* | Beta-glucosidase |
| CsaV3_5G000090 | *APRR5* | Two-component response regulator-like APRR5 |
| CsaV3_1G042560 | *BGLU12* | Beta-glucosidase 24 |
| CsaV3_3G045420 | *At3g50940* | ATP-dependent zinc metalloprotease FtsH-like protein |
| CsaV3_3G046460 | *SPX2* | SPX domain-containing protein |
| CsaV3_2G013640 | *CLH1* | Chlorophyllase-1-like |
| CsaV3_1G010300 | *CSE* | Caffeoylshikimate esterase-like |
| CsaV3_7G032820 | *MOT2* | Molybdate transporter like |
| CsaV3_1G002160 | *HIRL1* | Hypersensitive-induced response protein-like protein 1 |
| CsaV3_4G001170 | *PHOS32* | Universal stress protein A-like protein |
| CsaV3_6G048750 | *RBCX1* | Chaperonin-like RbcX protein |
| CsaV3_6G044780 | *ABCG6* | ABC transporter G family member 17-like |
| CsaV3_7G032050 | *FBL14* | F-box/LRR-repeat protein 14 |
| CsaV3_1G036140 | *AIRP2* | RING finger protein |
| CsaV3_5G031170 | *ABCB26* | Tetratricopeptide repeat (TPR)-like superfamily protein |
| CsaV3_5G006090 | *DJC76* | Chaperone protein DnaJ |
| CsaV3_3G045940 | *TLP1* | Thaumatin-like protein 1 |

**Table S7** Significantly changed metabolites mostly related to S fumigation in the leaves of cucumber plants in responses to S fumigation.

| Metabolites | VIP | *P*-value | FC | Post-hoc analysis (Fisher's LSD) |
| --- | --- | --- | --- | --- |
| L-Proline | 2.23 | 1.01E-02 | 1.30 | NSL0-vs-SL0 |
| L-Valine | 2.00 | 1.55E-02 | 0.46 | NSL12-vs-SL12 |
| L-Lysine | 1.91 | 2.23E-02 | 0.61 | NSL12-vs-SL12 |
| Sucrose | 13.89 | 9.76E-04 | 0.34 | NSL0-vs-SL0，NSL12-vs-SL12 |
| Trehalulose | 8.89 | 1.65E-04 | 0.31 | NSL0-vs-SL0 |
| 6-Kestose | 4.48 | 2.18E-02 | 0.40 | NSL0-vs-SL0 |
| D-Galactose | 4.44 | 2.24E-02 | 0.42 | NSL0-vs-SL0 |
| 3-Galactosyllactose | 3.23 | 1.12E-02 | 0.65 | NSL0-vs-SL0 |
| D-Glucose | 2.31 | 1.81E-03 | 0.78 | NSL0-vs-SL0 |
| 1-O-Feruloyl-β-D-glucose | 2.11 | 2.47E-02 | 0.42 | NSL0-vs-SL0 |
| 3-Keto-b-D-galactose | 1.64 | 3.31E-02 | 0.76 | NSL0-vs-SL0 |
| D-Fructose | 1.22 | 1.75E-02 | 0.83 | NSL0-vs-SL0 |
| UDP-L-rhamnose | 1.20 | 2.44E-02 | 0.27 | NSL0-vs-SL0 |
| Maltotetraose | 1.08 | 3.51E-02 | 0.45 | NSL0-vs-SL0 |
| Isovitexin 6''-O-glucoside | 35.11 | 9.43E-03 | 1.67 | NSL12-vs-SL12 |
| Isoscoparin 2''-O-glucoside | 12.10 | 3.08E-02 | 1.03 | NSL0-vs-SL0，NSL12-vs-SL12 |
| Cyanidin 3-glucogalactoside | 10.88 | 9.80E-03 | 1.57 | NSL12-vs-SL12 |
| Tectorigenin 7-O-gentiobioside | 9.86 | 3.35E-02 | 0.69 | NSL0-vs-SL0 |
| Nomilinic acid 17-glucoside | 2.52 | 4.53E-02 | 1.20 | NSL12-vs-SL12 |
| Phenylethyl primeveroside | 2.43 | 1.24E-02 | 1.39 | NSL12-vs-SL12 |
| Corchoionol C 9-glucoside | 1.79 | 9.37E-04 | 1.73 | NSL12-vs-SL12 |
| Calendulaglycoside E | 1.76 | 6.42E-04 | 2.21 | NSL12-vs-SL12 |
| Malvidin 3-rutinoside | 1.57 | 6.49E-03 | 0.79 | NSL0-vs-SL0 |
| Methyl helianthenoate F glucoside | 1.51 | 7.04E-03 | 0.67 | NSL0-vs-SL0 |
| Isorhamnetin 3-glucoside | 1.45 | 8.97E-03 | 2.44 | NSL12-vs-SL12 |
| Cinncassiol D4 2-glucoside | 1.44 | 8.02E-03 | 0.24 | NSL0-vs-SL0 |
| Petunidin 3-galactoside | 1.35 | 6.30E-04 | 2.60 | NSL12-vs-SL12 |
| Alliosterol 1-rhamnoside 16-galactoside | 1.23 | 1.70E-03 | 0.53 | NSL0-vs-SL0 |
| Methyl beta-D-glucopyranoside | 1.14 | 4.11E-02 | 0.38 | NSL0-vs-SL0 |
| 17-hydroxy-linolenic acid | 7.69 | 8.41E-03 | 0.92 | NSL0-vs-SL0，NSL12-vs-SL12 |
| Citric acid | 6.73 | 3.41E-02 | 1.48 | NSL0-vs-SL0 |
| Metabolites | VIP | *P*-value | FC | Post-hoc analysis (Fisher's LSD) |
| Pyrrolidonecarboxylic acid | 6.33 | 6.68E-03 | 1.12 | NSL0-vs-SL0，NSL12-vs-SL12 |
| Avenoleic acid | 4.91 | 4.71E-03 | 0.50 | NSL12-vs-SL12 |
| Isanic acid | 4.61 | 2.51E-03 | 0.43 | NSL12-vs-SL12 |
| Athanacalvic acid | 3.04 | 2.91E-03 | 0.59 | NSL12-vs-SL12 |
| Itaconic acid | 2.77 | 5.95E-03 | 0.62 | NSL12-vs-SL12 |
| 12-Keto-9Z,13Z,15Z-octadecatrienoic acid | 2.58 | 6.31E-04 | 0.42 | NSL12-vs-SL12 |
| 13-epi-12-oxo Phytodienoic Acid | 2.44 | 6.10E-03 | 0.59 | NSL12-vs-SL12 |
| 6-[5]-ladderane-hexanoic acid | 2.44 | 6.96E-04 | 0.56 | NSL12-vs-SL12 |
| Liquoric acid | 2.39 | 8.95E-03 | 0.62 | NSL12-vs-SL12 |
| Oxoglutaric acid | 2.32 | 3.81E-03 | 0.45 | NSL12-vs-SL12 |
| 12-tetradecynoic acid | 2.18 | 1.30E-02 | 0.29 | NSL12-vs-SL12 |
| Norophthalmic acid | 2.11 | 3.15E-02 | 3.09 | NSL0-vs-SL0 |
| Lucuminic acid | 1.92 | 6.55E-03 | 0.36 | NSL12-vs-SL12 |
| Ganoderic acid H | 1.92 | 2.65E-02 | 0.69 | NSL12-vs-SL12 |
| N-Acetylaspartylglutamic acid | 1.91 | 1.97E-02 | 0.61 | NSL12-vs-SL12 |
| L-Aspartic Acid | 1.84 | 1.83E-02 | 2.07 | NSL12-vs-SL12 |
| Isonicotinic acid | 1.88 | 2.72E-02 | 1.52 | NSL0-vs-SL0 |
| Alpha-kamlolenic acid | 1.70 | 3.89E-02 | 1.73 | NSL0-vs-SL0 |
| 4-Hydroxycinnamic acid | 1.24 | 1.51E-03 | 1.98 | NSL0-vs-SL0 |

**Table S8** Significantly changed metabolites mostly related to S fumigation in the fruits of cucumber plants in responses to S fumigation.

| Metabolites | VIP | P-value | FC | Post-hoc analysis (Fisher's LSD) |
| --- | --- | --- | --- | --- |
| L-Asparagine | 2.87 | 1.97E-02 | 0.38 | NSF0-vs-SF0 |
| D-Galactose | 4.45 | 1.88E-02 | 0.70 | NSF0-vs-SF0 |
| D-Glucose | 3.58 | 4.01E-02 | 0.75 | NSF0-vs-SF0 |
| 3-Galactosyllactose | 2.89 | 6.53E-03 | 0.28 | NSF0-vs-SF0 |
| D-(+)-Raffinose | 2.68 | 4.14E-02 | 0.51 | NSF0-vs-SF0 |
| D-Fructose | 2.07 | 2.64E-03 | 0.77 | NSF0-vs-SF0 |
| D-1,5-Anhydrofructose | 1.78 | 3.84E-02 | 0.79 | NSF0-vs-SF0 |
| (E)-1-O-Cinnamoyl-beta-D-glucose | 1.54 | 9.55E-05 | 0.08 | NSF0-vs-SF0 |
| Chinenoside I | 4.66 | 4.27E-02 | 0.79 | NSF0-vs-SF0 |
| Isorhamnetin 3-glucoside | 4.46 | 1.97E-02 | 0.68 | NSF0-vs-SF0 |
| Isovitexin 6''-O-glucoside | 2.93 | 4.86E-02 | 1.67 | NSF12-vs-SF12 |
| Lansioside B | 2.84 | 1.01E-02 | 0.51 | NSF0-vs-SF0 |
| Citroside A | 2.54 | 1.43E-03 | 0.54 | NSF0-vs-SF0 |
| Kurilensoside J | 2.29 | 4.37E-02 | 0.50 | NSF0-vs-SF0 |
| Sagittatoside A | 1.70 | 3.13E-02 | 0.28 | NSF0-vs-SF0 |
| Marmesin rutinoside | 1.50 | 3.07E-03 | 0.62 | NSF0-vs-SF0 |
| Corchoionoside B | 1.32 | 1.18E-02 | 0.61 | NSF0-vs-SF0 |
| Torvoside A | 1.32 | 3.85E-02 | 0.58 | NSF0-vs-SF0 |
| Cyanidin 3-glucogalactoside | 1.31 | 4.00E-02 | 1.78 | NSF12-vs-SF12 |
| Methyl helianthenoate F glucoside | 1.30 | 4.32E-02 | 0.70 | NSF0-vs-SF0 |
| Acuminoside | 1.25 | 4.28E-02 | 1.90 | NSF12-vs-SF12 |
| 2,3-Butanediol glucoside | 1.21 | 2.55E-02 | 0.32 | NSF0-vs-SF0 |
| Linalool 3,7-oxide beta-primeveroside | 1.17 | 3.52E-02 | 0.86 | NSF0-vs-SF0 |
| Prenyl arabinosyl-(1->6)-glucoside | 1.14 | 4.94E-03 | 0.74 | NSF0-vs-SF0 |
| 1-Hexanol arabinosylglucoside | 1.13 | 4.71E-03 | 0.54 | NSF0-vs-SF0 |
| Cynaroside A | 1.13 | 1.81E-02 | 0.43 | NSF0-vs-SF0 |
| Phenylethyl primeveroside | 1.05 | 4.40E-02 | 0.70 | NSF0-vs-SF0 |
| D8'-Merulinic acid A | 9.82 | 2.13E-03 | 1.28 | NSF0-vs-SF0 |
| Phosphohydroxypyruvic acid | 7.41 | 1.96E-03 | 1.66 | NSF0-vs-SF0 |
| Maleic acid | 4.65 | 3.43E-04 | 3.93 | NSF0-vs-SF0 |
| 9-tridecynoic acid | 4.25 | 1.51E-02 | 0.88 | NSF12-vs-SF12 |
| Metabolites | VIP | P-value | FC | Post-hoc analysis (Fisher's LSD) |
| Dodecanoic acid | 2.66 | 2.32E-03 | 0.66 | NSF12-vs-SF12 |
| 18-hydroxy stearic acid | 2.46 | 1.04E-02 | 1.52 | NSF0-vs-SF0 |
| Isonicotinic acid | 2.17 | 5.44E-03 | 1.36 | NSF0-vs-SF0 |
| Petroselinic acid | 1.99 | 1.52E-05 | 0.55 | NSF12-vs-SF12 |
| 9-tetradecynoic acid | 1.83 | 2.52E-02 | 1.98 | NSF0-vs-SF0 |
| Trihydroxycoprostanoic acid | 1.49 | 1.66E-02 | 1.09 | NSF0-vs-SF0, NSF12-vs-SF12 |
| 5E,7E-dodecadienoic acid | 1.76 | 2.12E-03 | 1.75 | NSF0-vs-SF0 |
| Elaidic Acid | 1.72 | 2.84E-04 | 0.52 | NSF12-vs-SF12 |
| Allantoic acid | 1.63 | 9.44E-04 | 0.58 | NSF12-vs-SF12 |
| 5-methyl-tetradecanedioic acid | 1.59 | 3.93E-02 | 1.90 | NSF0-vs-SF0 |
| Dodecanedioic acid | 1.55 | 4.92E-02 | 2.97 | NSF0-vs-SF0 |
| 9-hydroxy-12-octadecenoic acid | 1.50 | 1.85E-04 | 0.68 | NSF12-vs-SF12 |
| 5-Heptyltetrahydro-2-oxo-3-furancarboxylic acid | 1.49 | 9.37E-03 | 1.88 | NSF0-vs-SF0 |
| Myristic acid | 1.49 | 3.79E-02 | 1.58 | NSF0-vs-SF0 |
| Aleprylic acid | 1.46 | 4.26E-02 | 0.87 | NSF12-vs-SF12 |
| cis-7-Hexadecenoic Acid | 1.44 | 5.28E-04 | 0.67 | NSF12-vs-SF12 |
| cis-10-palmitoleic acid | 1.35 | 8.64E-04 | 0.66 | NSF12-vs-SF12 |
| 15-hydroxy-pentadecanoic acid | 1.31 | 2.11E-02 | 2.39 | NSF0-vs-SF0 |
| 4-Formylsalicylic acid | 1.17 | 8.60E-03 | 0.83 | NSF12-vs-SF12 |
| Lycoperdic acid | 1.15 | 4.24E-02 | 1.64 | NSF0-vs-SF0 |
| Norophthalmic acid | 1.15 | 4.98E-03 | 2.76 | NSF0-vs-SF0 |
| 10,20-Dihydroxyeicosanoic acid | 1.13 | 1.02E-02 | 0.76 | NSF12-vs-SF12 |
| omega-hydroxy myristic acid | 1.10 | 7.40E-03 | 0.58 | NSF12-vs-SF12 |
| 9-Pentadecenoic acid | 1.09 | 5.14E-04 | 1.34 | NSF0-vs-SF0 |
| 2-isopropyl-malic acid | 1.06 | 4.99E-02 | 0.81 | NSF12-vs-SF12 |
| 8,13-dihydroxy-9,11-octadecadienoic acid | 1.05 | 1.06E-02 | 1.65 | NSF0-vs-SF0 |

**Table S9** Transcript loading scores from data integration analysis using O2PLS multivariate analytical methods.

| Gene ID | Loading_1 | Loading_2 |
| --- | --- | --- |
| CsaV3_2G013360 | 0.1586 | -0.2951 |
| CsaV3_1G042860 | -0.1984 | 0.1971 |
| CsaV3_4G000200 | 0.0581 | 0.2724 |
| CsaV3_6G038720 | -0.1263 | -0.2460 |
| CsaV3_4G027320 | 0.1820 | -0.1977 |
| CsaV3_2G001740 | 0.0610 | 0.2502 |
| CsaV3_3G041150 | 0.0502 | -0.2444 |
| CsaV3_2G001760 | 0.1795 | -0.1688 |
| CsaV3_3G043020 | 0.2136 | -0.1175 |
| CsaV3_3G045740 | -0.0602 | 0.2331 |
| CsaV3_2G001750 | 0.0954 | 0.2142 |
| CsaV3_7G031490 | -0.2060 | -0.1040 |
| CsaV3_1G013880 | -0.1833 | -0.1381 |
| CsaV3_5G030130 | -0.1460 | -0.1768 |
| CsaV3_1G039210 | 0.1798 | 0.1339 |
| CsaV3_7G018620 | -0.2204 | -0.0229 |
| CsaV3_6G049640 | -0.1873 | 0.1125 |
| CsaV3_6G045790 | -0.2093 | 0.0074 |
| CsaV3_5G000090 | -0.1266 | -0.1657 |
| CsaV3_2G029720 | 0.1976 | 0.0351 |
| CsaV3_4G024430 | -0.1539 | -0.1211 |
| CsaV3_6G048960 | -0.1320 | 0.1426 |
| CsaV3_5G004860 | -0.1729 | -0.0742 |
| CsaV3_7G025390 | 0.1689 | 0.0778 |
| CsaV3_4G013230 | -0.1620 | 0.0900 |
| CsaV3_6G005910 | -0.1671 | -0.0786 |
| CsaV3_3G002030 | 0.1442 | 0.1139 |
| CsaV3_3G011770 | 0.1426 | 0.1148 |
| CsaV3_6G031510 | -0.1275 | 0.1274 |
| CsaV3_3G012820 | -0.0824 | 0.1574 |
| CsaV3_3G018980 | 0.1574 | 0.0773 |
| Gene ID | Loading_1 | Loading_2 |
| CsaV3_3G043250 | -0.1090 | -0.1364 |
| CsaV3_4G032640 | 0.1526 | 0.0822 |
| CsaV3_2G013530 | 0.1590 | -0.0531 |
| CsaV3_1G017010 | 0.1609 | 0.0407 |
| CsaV3_1G010690 | -0.0099 | 0.1643 |
| CsaV3_1G005680 | 0.0936 | -0.1248 |
| CsaV3_3G049490 | 0.0761 | 0.1328 |
| CsaV3_2G017890 | 0.1502 | 0.0098 |
| CsaV3_3G044460 | 0.1466 | -0.0103 |
| CsaV3_7G033120 | -0.1055 | 0.0954 |
| CsaV3_3G004900 | -0.1385 | -0.0278 |
| CsaV3_3G011170 | -0.0919 | 0.0962 |
| CsaV3_3G012960 | -0.0931 | 0.0820 |
| CsaV3_2G001680 | -0.0530 | -0.1069 |
| CsaV3_3G039700 | 0.1123 | -0.0329 |
| CsaV3_1G030670 | -0.0422 | 0.1086 |
| CsaV3_5G013270 | 0.0782 | -0.0756 |
| CsaV3_7G027830 | -0.0792 | 0.0713 |
| CsaV3_6G044100 | -0.0444 | 0.0955 |
| CsaV3_4G024220 | -0.0336 | 0.0997 |
| CsaV3_6G047800 | 0.0437 | -0.0562 |
| CsaV3_5G023720 | -0.0221 | -0.0583 |
| CsaV3_2G034140 | 0.0512 | -0.0330 |
| CsaV3_5G026240 | 0.0319 | -0.0308 |
| CsaV3_5G002830 | -0.0024 | -0.0404 |
| CsaV3_1G030710 | -0.0099 | 0.0079 |

**Table S10** Metabolite loading scores from data integration analysis using O2PLS multivariate analytical methods.

| Metabolites | Loading_1 | Loading_2 |
| --- | --- | --- |
| Nicotianamine | 0.1555 | 0.4948 |
| S-Adenosylmethionine | 0.4494 | -0.1896 |
| L-Cystine | -0.3729 | -0.3035 |
| Sulfate | -0.4292 | -0.0327 |
| 3-Sulfinoalanine | -0.4187 | -0.0380 |
| L-Threonine | -0.0944 | 0.4022 |
| S-Methylmethionine | 0.2400 | -0.3286 |
| L-Cystathionine | 0.2181 | 0.2929 |
| Homocysteine | -0.0817 | -0.3471 |
| Glutathione | 0.2307 | -0.2705 |
| O-Phosphohomoserine | 0.2570 | -0.0225 |
| L-Methionine | -0.1747 | 0.1491 |
| 1-Aminocyclopropanecarboxylic acid | 0.0543 | -0.1744 |
| L-Serine | -0.0439 | 0.1494 |
