# Supplementary material for: The Transcriptome and Metabolome Reveal Stress Responses in Sulfur-Fumigated Cucumber (Cucumis sativus L.)
Source: Front Plant Sci. 2021 Nov 12;12:778956. doi: 10.3389/fpls.2021.778956 (PMC8636124; doi:10.3389/fpls.2021.778956)
Supplement: Supplementary file 2 [file Data_Sheet_2.docx]

**
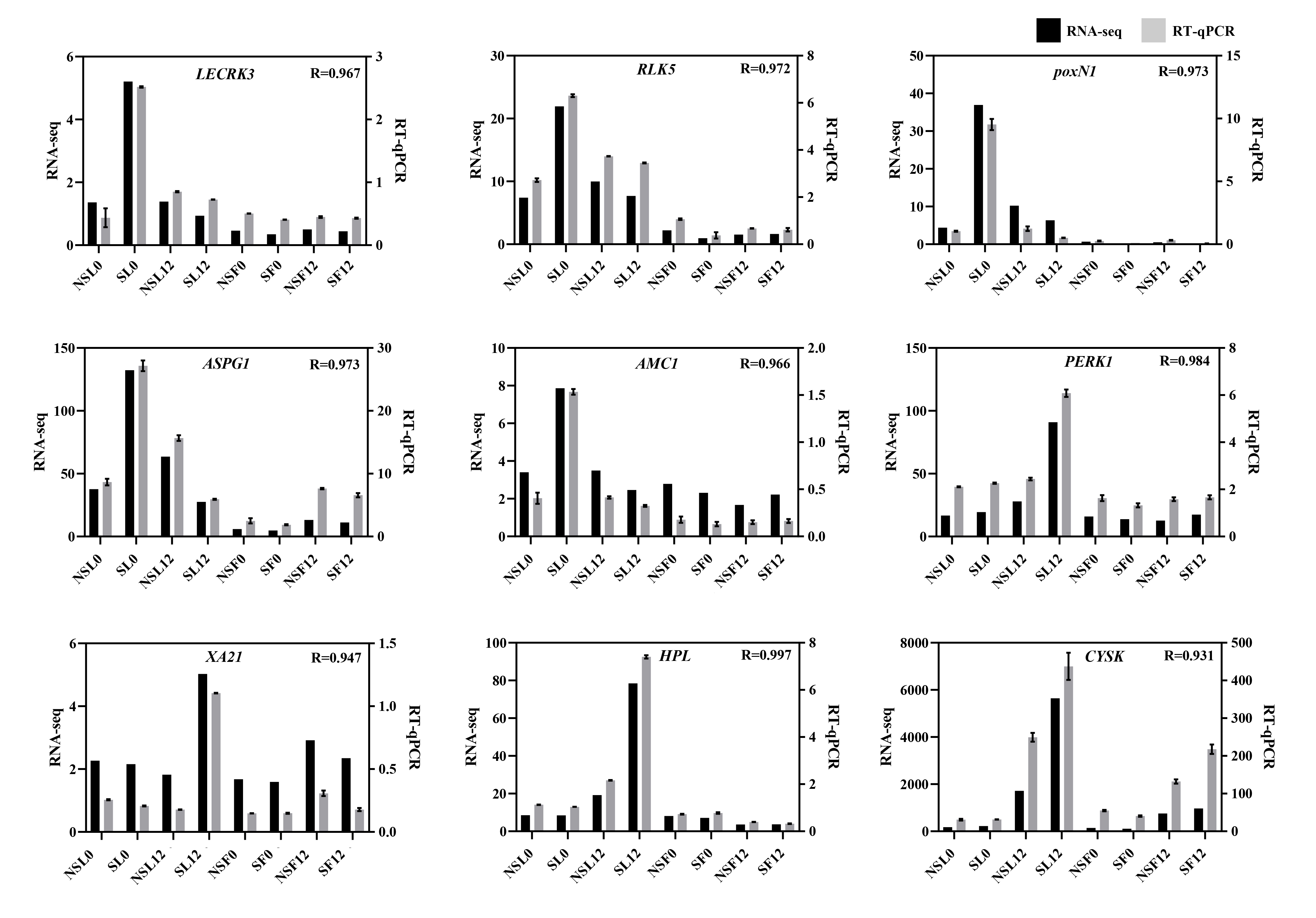
**

**Figure S1** RT-qPCR verified hub genes related to stress response. Error bars represent standard deviation of three replicates.

**
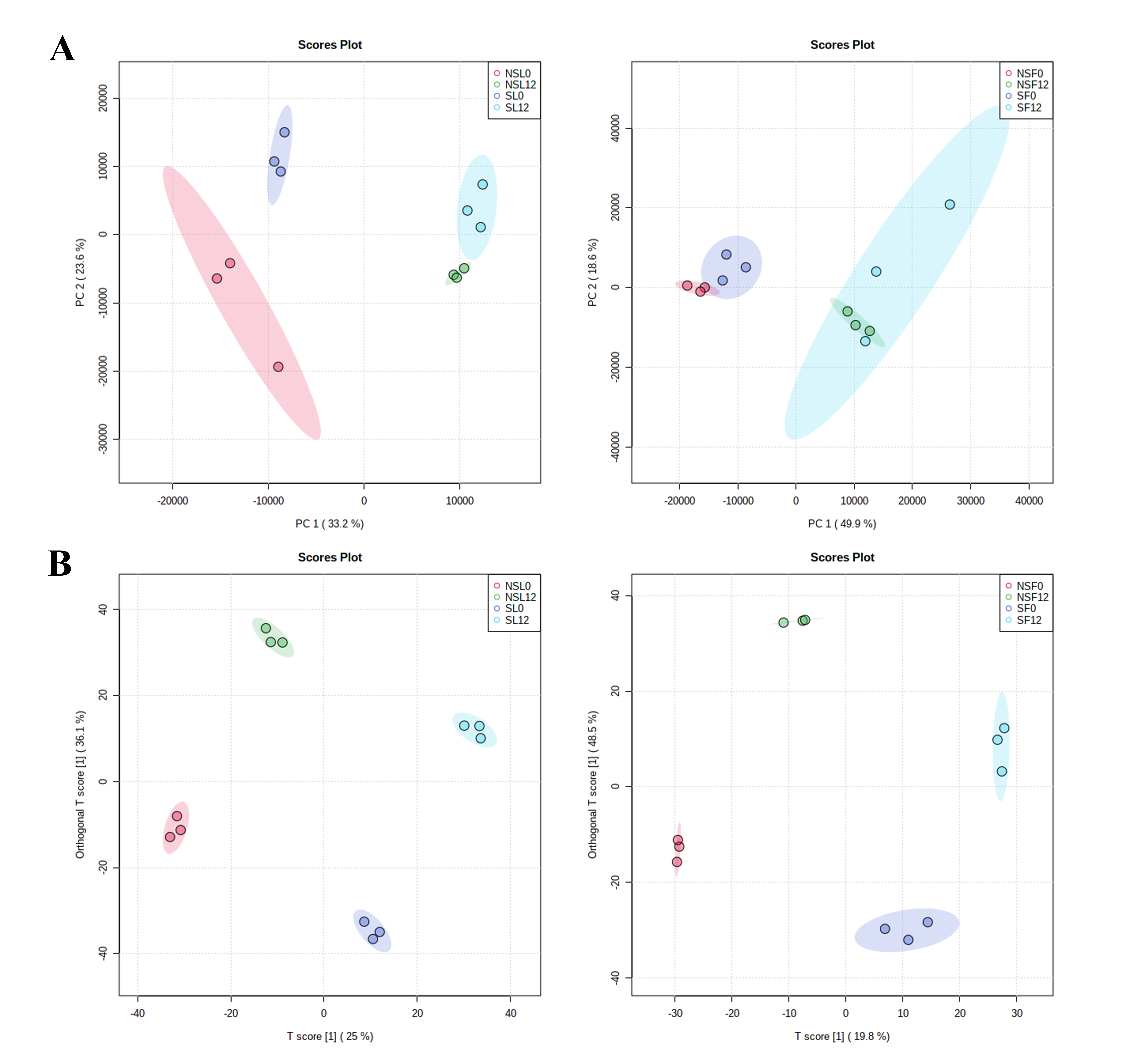
**

**Figure S2** PCA score plots (A) and (O)PLS-DA loading plots (B) of metabolites in the leaves and fruits of non-fumigated and S-fumigated treatments.

**
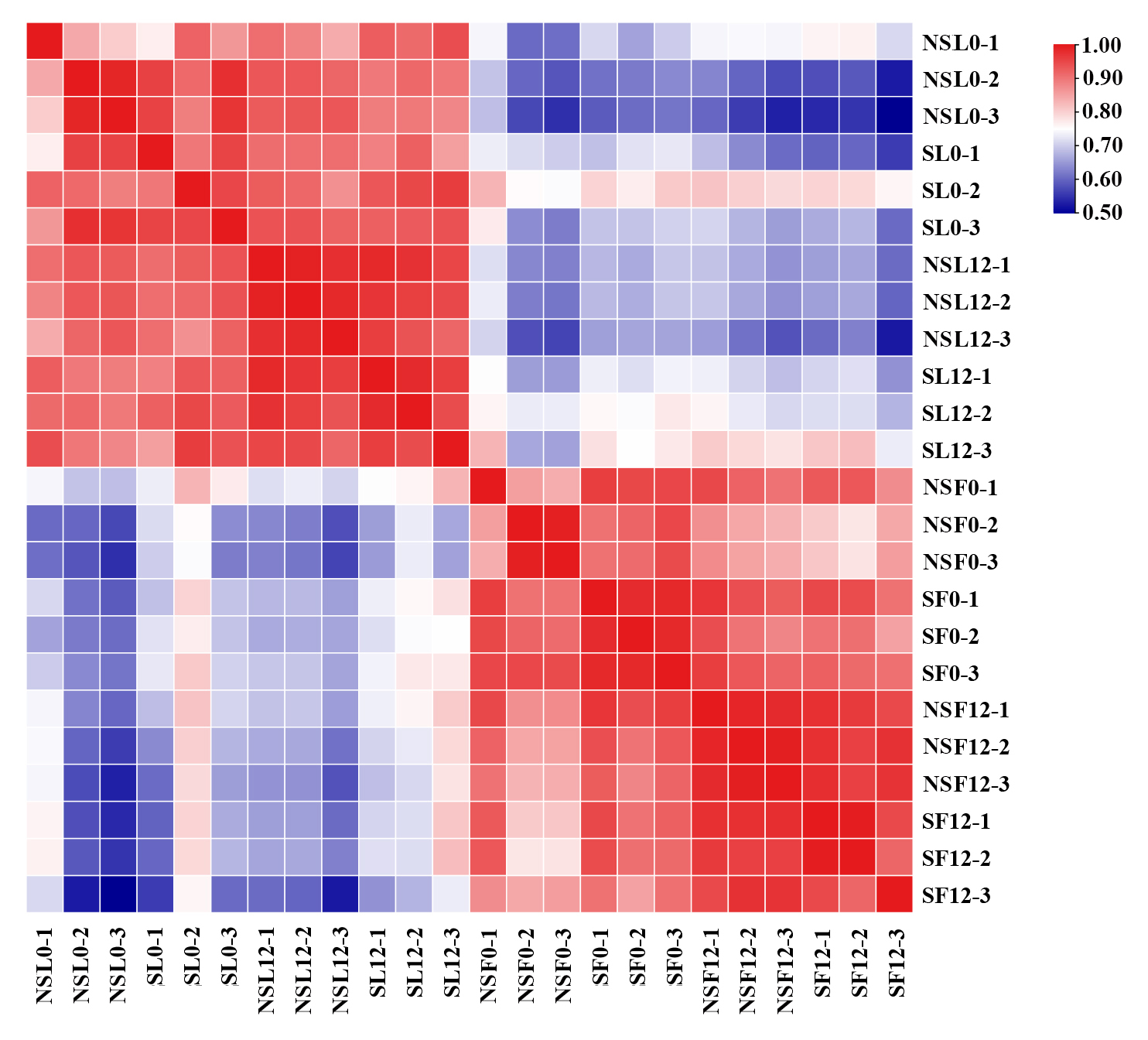
**

**Figure S3** Heatmap of Pearson’s correlation coefficient among L0, L12, F0, F12 from control and S fumigation, including replicates.


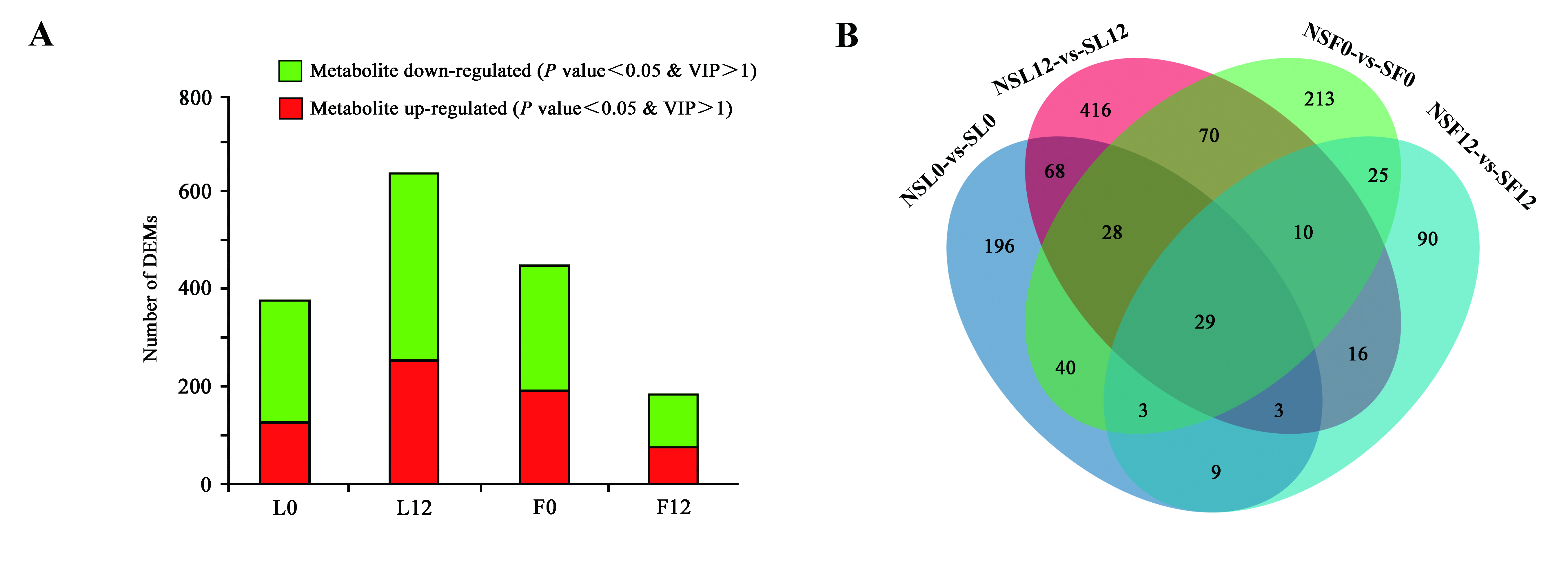


**Figure S4** The number and Venn diagram of differentially expressed metabolites (DEMs) between each group.
