# Supplementary material for: The Transcriptome and Metabolome Reveal Stress Responses in Sulfur-Fumigated Cucumber (Cucumis sativus L.)
Source: Front Plant Sci. 2021 Nov 12;12:778956. doi: 10.3389/fpls.2021.778956 (PMC8636124; doi:10.3389/fpls.2021.778956)
Supplement: Supplementary file 3 [file Data_Sheet_3.docx]

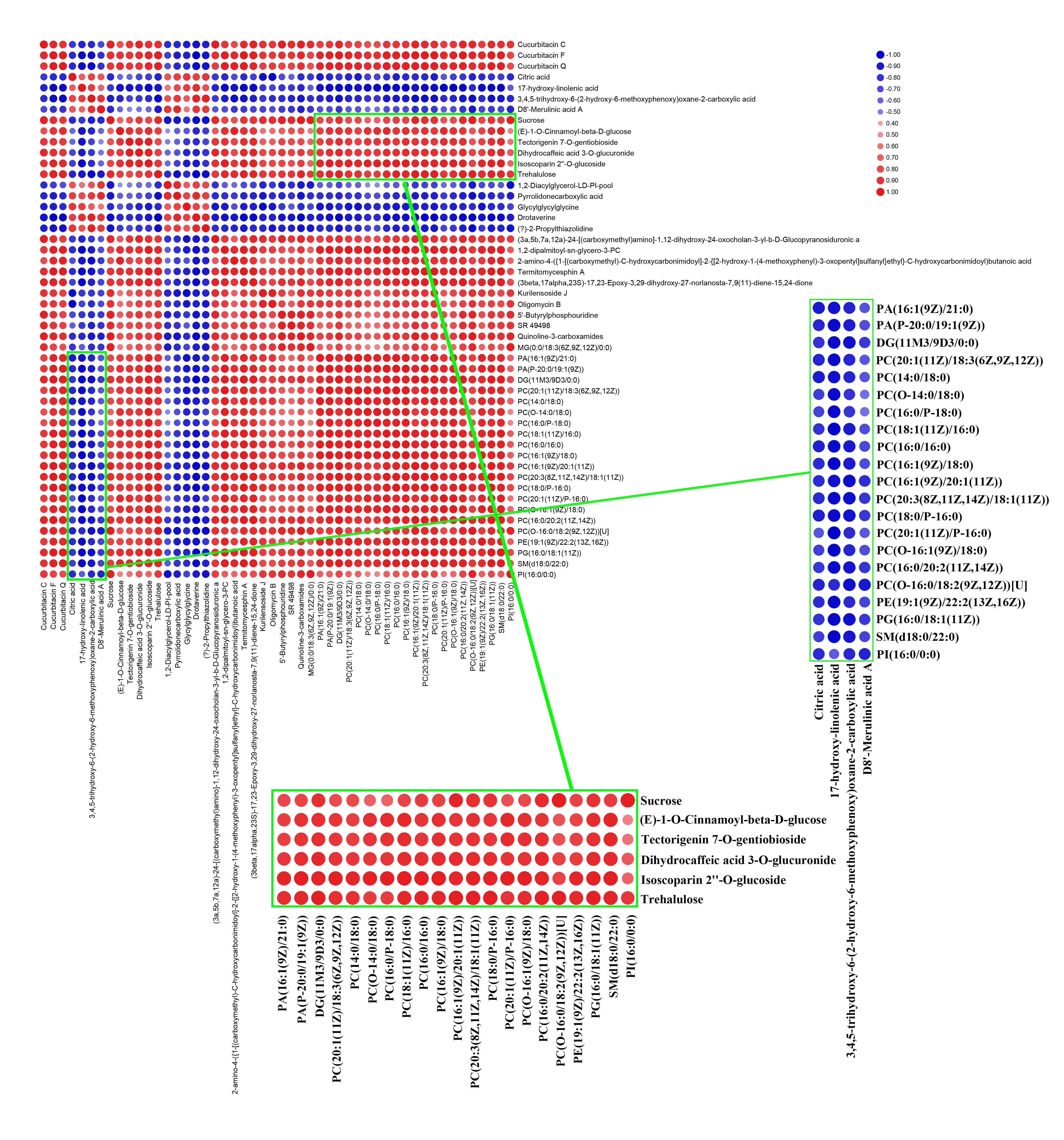


**Figure S5** Heatmap of the correlation between the top 50 VIP differentially expressed metabolites (DEMs) in L0 with S fumigation. Each square represents a Pearson’s correlation coefficient.


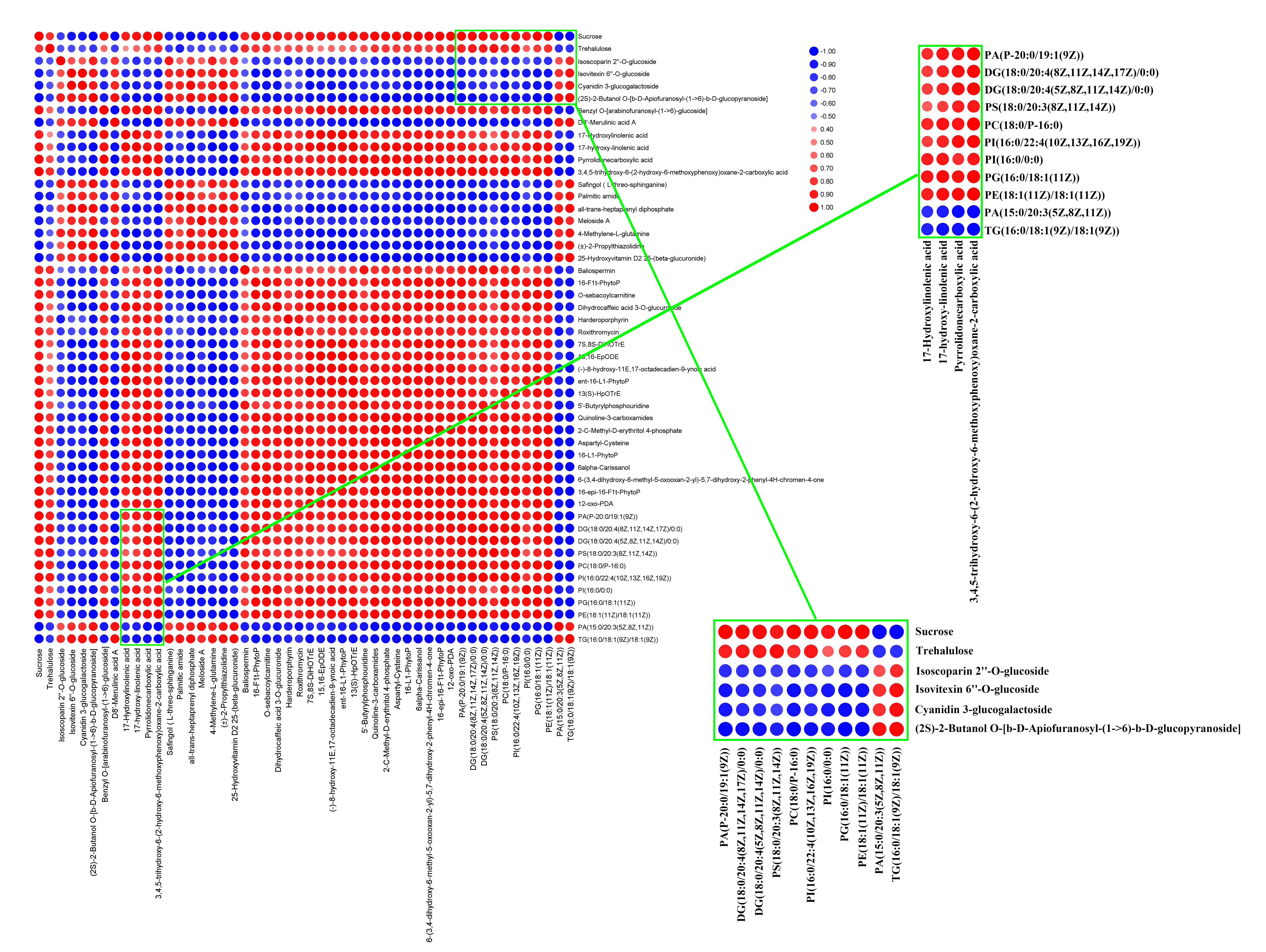


**Figure S6** Heatmap of the correlation between the top 50 VIP differentially expressed metabolites (DEMs) in L12 with S fumigation. Each square represents a Pearson’s correlation coefficient.
